# Testing evolutionary theories of human cooperation via meta-analysis of microfinance repayment

**DOI:** 10.1017/ehs.2026.10047

**Published:** 2026-04-06

**Authors:** Dugald Foster, Erik Postma, Shakti Lamba, Alex Mesoudi

**Affiliations:** Centre for Ecology and Conservation, University of Exeter Cornwall Campus, Penryn, UK

**Keywords:** cooperation, punishment, microfinance, meta-analysis, social dilemma’

## Abstract

Explaining how cooperation evolves is a major research programme in the biological and social sciences. In this study, we tested evolutionary theories of human cooperation in a real-world social dilemma: joint liability microfinance, in which groups of borrowers must cooperate to successfully repay a shared loan. We used pre-registered Bayesian multilevel models to estimate meta-analytic associations between loan repayment and proxies of four evolutionary mechanisms proposed to support cooperation: relatedness, reciprocity, partner choice, and punishment. A systematic search of the microfinance literature yielded 73 effect estimates for 11 proxies of evolutionary mechanisms analysed in 11 separate meta-analyses. Punishment-based variables showed the strongest positive meta-analytic associations with loan repayment, with mixed results for other mechanisms. However, estimates varied widely in their certainty, with generally high levels of between-study heterogeneity. Our results provide some evidence for evolutionary mechanisms supporting cooperation in real-world contexts, but also indicate there are non-generalisable findings and/or reproducibility issues in the microfinance literature.

## Graphical abstract

**Figure:**
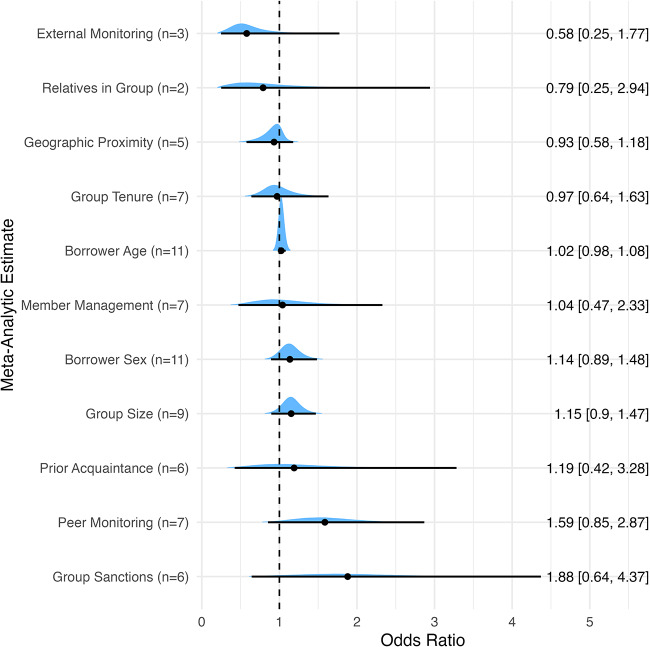

## Social media summary

Meta-analyses reveal the evolutionary mechanisms that encourage cooperation in microfinance loan repayment groups

## Introduction

Researchers have identified several mechanisms capable of supporting the evolution and maintenance of cooperation in humans and other species (Apicella & Silk, 2019; Fletcher & Michael Doebeli, 2009; Lehmann & Keller, 2006; Nowak, 2006; West et al., 2007a; West et al., 2007b), and which could explain why humans are more or less likely to cooperate under particular circumstances. Theoretical models, laboratory experiments, and field studies have all been used to explore the viability of these mechanisms in promoting cooperation (Apicella & Silk, 2019; Nowak, 2006). However, most studies have tested the effects of individual mechanisms in isolation, with few comparing mechanisms simultaneously using real-world human behavioural data. Due to its global implementation across diverse human ecologies, populations, and cultures, joint liability microfinance provides an opportunity to test multiple evolutionary mechanisms of cooperation, including kin selection, reciprocity, partner choice, and punishment, within the context of the same real-world social dilemma.

Microfinance loans are designed for people who cannot access traditional loans from banks, either because they are unable to provide collateral such as land or property required as a guarantee against non-repayment or because they are excluded from financial services due to geographic or economic barriers (Armendáriz & Morduch, 2010). Some microfinance institutions (MFIs) offer ‘joint liability’ loans (sometimes called ‘group loans’ or ‘solidarity loans’), provided to a group of borrowers who assume collective responsibility for repaying the loan. Once a group has been formed by individuals interested in taking out a loan, the group signs a loan contract with the lending institution stipulating the size of the loan, the interest rate, and the repayment schedule. Crucially, all members of the group must accept the terms of joint liability attached to the loan. Borrowers in the group then receive and invest their loans at their discretion. At agreed intervals, the group convenes at repayment meetings where members repay their individual shares of the loan to the lending institution. If a borrower does not attend the meeting or fails to repay his/her share, the other group members are collectively liable for his/her repayment. If the group fails to repay the loan in full, all members are banned from accessing further loans and could face additional penalties (Armendáriz & Morduch, 2010).

Prominent theoretical models in economics assume that joint liability loans allow MFIs to insure themselves against the risk of individual borrowers failing to repay, by replacing material collateral with social collateral (Armendáriz, 1999; Besley & Coate, 1995; Conning & Udry, 2007; Ghatak & Guinnane, 1999; Stiglitz, 1990; Van Tassel, 1999; Varian, 1990). These models highlight that the shared responsibility of repaying a group loan creates a social dilemma: a scenario in which ‘selfish’ individuals who maximise their own success outperform cooperative individuals who pay a cost for the sake of the group, but where groups of cooperators outperform groups of non-cooperators (Dawes, 1980; Olson, 1971). Hence, individual- and group-level interests conflict. Under joint liability, microfinance borrowers seeking to maximise their immediate financial returns have an incentive to default on their repayment dues, in effect receiving a free loan, and to free-ride on the repayment efforts of others in their loan group. Yet the loan group succeeds only if the total loan amount is repaid, thereby ensuring continued access to future and potentially larger loans for all group members. To successfully repay a group loan, borrowers must therefore cooperate: first by repaying their own share of the loan, and if necessary by enforcing the payment of money owed by any defaulting group members, or else paying it themselves (Besley & Coate, 1995).

Microfinance loans are popular globally, with around 140 million customers borrowing 125 billion US dollars in 2018, and with large markets operating in Southeast Asia, sub-Saharan Africa, Latin America and the Caribbean, and East Asia and the Pacific (Microfinance Barometer, 2019). Given the popularity of microfinance, and because of the mixed evidence for its success in poverty reduction (Meager, 2019; Roodman, 2012), it is important to understand what prevents loan groups from becoming overrun by free-riding defaulters. While many MFIs regularly report high repayment rates for group loans (Cull et al., 2007), there is considerable variation in loan repayment efficacy across time and space (Attanasio et al., 2015; Carpena et al., 2013; Giné & Karlan, 2014; Mahmud, 2019). For example, the Grameen Bank, one of the largest MFIs in Bangladesh, has reported variation in repayment rates for group loans from 100% of loans repaid in one district to 30% in another (Woolcock, 1999). During the Indian microfinance crisis of 2010, national group loan repayment rates dropped from 98% to 10% before subsequently recovering (Haldar & Stiglitz, 2016). We hypothesise that this variation in repayment rates reflects variation in the ability of loan groups to solve the social dilemma underlying the joint liability model. We therefore predict that if joint liability lending relies on cooperation between borrowers, factors that facilitate cooperation between borrowers will show a positive association with group loan repayment efficacy.

Lamba (2014) first identified the potential of using joint liability lending as a model system to test evolutionary theories of cooperation. This was developed by Gehrig et al. (2021), who qualitatively summarised the effects of a range of variables representing evolutionary mechanisms of cooperation on loan repayment. Surprisingly, genetic relatedness between group members was found to be largely negatively associated with repayment efficacy, although prior interaction (i.e. reciprocity) and partner choice were both more positively associated. As noted by Gehrig et al. (2021), however, the vote counting method they used, in which statistically significant findings were tallied across studies, has severe limitations, including an inability to assess the magnitude of effects and a failure to assign differential weights to studies based on characteristics such as study quality and statistical power (Higgins & Thomas, 2019). Nonetheless, Gehrig et al. (2021) provided a useful preliminary evaluation of the loan repayment literature as a model system to test evolutionary theories of cooperation, which merits further investigation.

Here we conduct quantitative, theoretically informed, and pre-registered meta-analyses of predictors of joint liability loan repayment to estimate average associations between repayment and proxies of evolutionary mechanisms of cooperation and to assess the uncertainty, heterogeneity, and generalisability of these estimates. We find weak and largely inconsistent evidence of associations between our evolutionarily informed predictors and loan repayment, with substantial uncertainty in our estimates predominantly caused by a large degree of between-study heterogeneity that limits the generalisability of our findings. However, in conducting our meta-analyses, we identify several limitations in the existing microfinance literature that generate constraints in terms of causal inference, including inconsistent measurement of effect estimates and predictor and outcome variables, endogeneity in loan group formation, uncontrolled variation in study design and settings, and over-reliance on cross-sectional, non-experimental designs.

### Study hypotheses and predictions

We tested four hypotheses based on prominent evolutionary mechanisms that could plausibly support cooperation among microfinance borrowers. These mechanisms all act by increasing assortment between cooperators so that cooperators are more likely to interact with other cooperators, and less so with free-riders (Fletcher & Michael Doebeli, 2009). We originally planned to test three more hypotheses (see study pre-registration at https://osf.io/9e6ay), but the microfinance literature we reviewed was unable to provide the necessary data. For each hypothesised mechanism, we make predictions regarding its effect on group loan repayment outcomes. We declare no predictions concerning the relative magnitude of the effects of these mechanisms, which may interact in complex ways.

### Kin selection via common ancestry

Loan groups composed of more relatives/more closely related borrowers should exhibit higher repayment rates compared to groups composed of fewer relatives/less closely related borrowers. Theoretical models demonstrate that cooperation can evolve when it is preferentially directed towards genetic relatives who are more closely related to the cooperator due to their common ancestry, relative to the population mean (Hamilton, 1964a, 1964b). Cooperative behaviours that evolve due to their positive impacts on the success of relatives despite their costs to the actor are said to result from ‘kin selection’ (Grafen, 2007; Maynard Smith, 1964). Given some genetic basis to cooperative behaviour and a human evolutionary history of cooperation driven by kin selection, assortment between relatives could correspond to assortment between cooperators. In joint liability loan groups composed of genetic relatives, the benefits provided to relatives by successful repayment could therefore outweigh the costs of cooperation.

### Prior interaction and reciprocity

Loan groups composed of borrowers with experience or knowledge of one another’s past cooperative behaviour should exhibit higher repayment rates. Cooperation can evolve when it is preferentially directed towards individuals known to be cooperators based on their past behaviour (Trivers, 1971). Direct reciprocity describes an actor’s decision to cooperate with an individual who has previously cooperated with that actor (Axelrod & Hamilton, 1981; Trivers, 1971). Indirect reciprocity describes an actor’s decision to cooperate based on their knowledge of prior interactions between an individual and others, or by using information regarding that individual’s reputation for cooperation (Nowak & Sigmund, 1998). In the context of microfinance, measures of prior interaction amongst group members such as whether group members knew each other before forming the group, the length of time the group has been in existence, the frequency of group meetings, and the geographical proximity of group members should all provide information about other group members’ past histories of cooperation and therefore affect repayment via reciprocity. For example, group members might choose to help defaulters repay a loan when they themselves have previously been the beneficiary of repayment assistance from that defaulter (direct reciprocity). Or, they may help a defaulter repay a loan because they know that this defaulter has always paid off their loans in the past and this failure to repay is a one-off event rather than a repeated pattern of free-riding (indirect reciprocity). While prior interactions might also facilitate the *failure* to repay a loan if those defaulters are known instead to be free-riders, such instances are likely to be rarer, as failure to repay loans will preclude continued access to subsequent loans. In any case, free-riding is easier when there is a lack of information about past cooperation, hence our prediction that more frequent prior interaction should on average increase repayment rates.

### Partner choice

Loan groups formed by borrowers themselves should exhibit higher repayment rates compared to groups formed by a lending institution or groups formed at random. Previous work has shown that cooperation can evolve when cooperators can choose to preferentially interact with other known cooperators and actively dissociate from free-riders, leading to positive assortment (Aktipis, 2004; Hruschka & Henrich, 2006; McNamara et al., 2008; Noë & Hammerstein, 1994). While reciprocity describes decisions to cooperate given the partners available once a group has been formed (e.g. whether to help another group member repay their loan), it does not encompass the choice of available partners at the time of group formation. Selecting cooperative partners from the outset through partner choice may do more to facilitate cooperation than individual decisions made during future interactions (Hruschka & Henrich, 2006). While some loan groups are formed with input from the lending institution (Feigenberg et al., 2013; Karlan, 2007), others are formed by borrowers themselves (Banerjee et al., 2015; Morduch, 1999). Some MFIs also allow loan groups to alter their group membership, for example, by ejecting a persistently defaulting borrower (Carpenter & Williams, 2014), which could also increase assortment between cooperators.

### Punishment

Loan groups which carry out sanctions against defaulters, and those that conduct higher levels of monitoring, should exhibit higher repayment rates. Punishment has been identified as central to solving social dilemmas, particularly for large groups of unrelated individuals that have been formed (initially) without assortment (Ågren et al., 2019; Fehr & Gächter, 2002; Frank, 1995). By attaching an extra cost to free-riding, punishment can alter the relative costs and benefits of cooperating, so that cooperation offers the highest pay-offs (Lehmann & Keller, 2006), and would-be free-riders turn to cooperation, thereby increasing assortment. Studies of real-world collective action problems (Herrmann et al., 2008; Kaplan et al., 1985; Ostrom et al., 1992; Wiessner, 2005) and laboratory experiments (Ertan et al., 2009; Fehr & Gächter, 2002; Gürerk et al., 2006; Yamagishi, 1986) support the importance of punishment as a mechanism for maintaining cooperation. While policing free-riders and coordinating punishment may be associated with their own costs (Boyd & Richerson, 1992; Smaldino et al., 2013), cooperative punishers can be rewarded for their efforts through the receipt of additional benefits, for example, via the ostracism of free-riders from the group, resulting in an increased likelihood of interacting with other cooperators (Bowles & Gintis, 2004; Frank, 2003). Similarly, if the costs of punishment are shared between a sufficiently large proportion of group members, punishment is both more effective and the individual costs of delivering punishment decline (Boyd et al., 2010, 2003). While little is known about the dynamics of actual punishment within joint liability loan groups (Solli et al., 2015), there is anecdotal evidence of groups cooperating to punish defaulters, ranging from mild social shaming of defaulters to collectively confronting them at home, to seizing their household goods and demanding payment for their release (Solli et al., 2015).

### Summary of hypotheses

We pre-registered four primary predictions based on evolutionary mechanisms capable of supporting cooperation: relatedness, reciprocity, partner choice, and punishment. We also pre-registered exploratory meta-analyses of three variables that appear regularly in the loan repayment literature (Group size, Borrower age, and Borrower sex) but which do not represent discrete mechanisms or clearly align with any particular hypotheses. We did however have theoretical reasons to investigate these variables, all of which have been implicated in evolutionary theories of cooperation and assortment. For example, some models suggest that cooperative behaviour and reputations are harder to track in larger groups (Suzuki & Akiyama, 2005), and experiments have shown that the reduction in marginal returns with increasing group size can reduce the benefits of cooperation (Isaac & Walker, 1988). Depending on the method of group formation, larger groups could also require a greater proportion of cooperators to maintain cooperation, potentially creating an obstacle to the evolution of cooperation via reciprocity (Boyd & Richerson, 1988). We therefore predicted that larger loan groups would show worse repayment efficacy on average. We did not make directional predictions for borrower sex or age, but still planned to model their average effects, as this could provide insights of applied relevance to microfinance lending. We also tested the role of each of these three variables in explaining heterogeneity between studies.

## Methods

We used meta-analysis (Gurevitch et al., 2018; Moreau & Gamble, 2022; Quintana, 2015) to estimate overall associations between a range of variables and loan repayment. To inform our study, we conducted a pilot analysis of the 41 studies included in Gehrig et al. (2021) (see Supplementary Materials SM1). This pilot analysis qualitatively evaluated and compared the statistical models and variable types reported in the 41 studies from Gehrig et al. (2021) in order to develop a feasible statistical meta-analytic strategy (note that no formal meta-analyses were performed in the pilot study itself). Based on the pilot study, we selected 12 variables to meta-analyse (see Table 1). We chose these variables based on their frequent occurrence in group loan repayment studies and their suitability as proxies for the evolutionary mechanisms discussed above. Nine variables are directly linked to specific mechanisms, while three are ambiguous in their relationship to any particular mechanism but may influence the returns from cooperation (Table 1). We pre-registered our predictions and methods on the OSF platform, using the PRISMA-P template (Moher et al., 2015 ) to specify our methods, available at https://osf.io/3y9fu.

**Table 1.** Common predictor variables analysed in loan repayment studies and included in our meta-analysis, and their association with evolutionary mechanisms of cooperation

Variable	Evolutionary mechanism	Justification
**VC1.** Relatives in the loan group (binary)	Common ancestry	The presence of family members in loan groups provides an indication of genetic relatedness within groups.
**VC2.** Prior acquaintance of group members (binary)	Prior interaction	Borrowers who know each other prior to joining a group are more likely to have information about each other’s cooperative histories.
**VC3.** Group tenure (months)	Prior interaction	Borrowers belonging to more long-lasting loan groups are more likely to have information about each other’s cooperative histories.
**VC4.** Frequency of group meetings (weeks)	Prior interaction	Borrowers who meet more frequently are more likely to have information about each other’s cooperative histories.
**VC5.** Geographic proximity of group members (km)	Prior interaction	Borrowers who live or work closer to one another are more likely to have information about each other’s cooperative histories.
**VC6.** Management of group membership (binary)	Partner choice	Loan groups which actively manage their membership (e.g. by screening potential members) enable the selection of cooperative partners.
**VC7.** Group sanctions (binary)	Punishment	Loan groups which impose their own penalties on defaulters create additional costs for non-repayment.
**VC8.** Peer monitoring (binary)	Punishment	Borrowers who experience higher levels of monitoring by their loan group are more likely to increase their repayment effort to avoid punishment.
**VC9.** External monitoring (binary)	Punishment	Borrowers who experience higher levels of monitoring by the loan officer or lending institution are more likely to increase their repayment effort to avoid punishment.
**VC10.** Group size	N/A	No particular mechanism
**VC11.** Borrower age (years)	N/A	No particular mechanism
**VC12.** Borrower sex	N/A	No particular mechanism

*Note*: ‘Binary’ indicates that the predictor is of the form ‘yes/no’ or ‘present/absent’, otherwise units are provided for continuous predictors.

### Meta-analysis eligibility criteria

Studies had to meet three criteria to be eligible for inclusion in the meta-analyses:

1. *Relevant predictor variables and outcome measure*

Studies had to report quantitative analysis of a predictor variable relevant to 1 of the 12 variables we planned to meta-analyse (Table 1), and its association with an outcome measure of joint liability loan repayment. This includes experimental and cross-sectional studies of group loan repayment and studies comparing repayment between group and individual loans.

1. *Publication status*

Studies must have undergone peer review or some form of expert assessment, including scientific journal publications, unpublished but examined doctoral theses, and working papers prepared for conferences or journal submission. Here, we aimed to strike a balance between ensuring a high quality of studies and including relevant results, which may exist outside journal publications. Any study that did not meet quality criteria as measured by our risk of bias threshold (see below) was excluded from our main analyses.

1. *Years and languages considered*

We considered all English-language studies available online before July 2021.

### Information sources

For the primary literature search, we used Google Scholar, Web of Science, EconLit, and ProQuest search engines, with the aim of improving search performance by combining results from multiple databases and multiple search engines (Bramer et al., 2017). To check for eligible papers missed in our primary search, we also hand-searched the reference lists of eligible studies and used the CoCites literature search tool (Janssens et al., 2020) to identify the most frequently co-cited papers of the most relevant publications as defined by our inclusion criteria.

### Search strategy

Search terms are presented in Table S1a. In addition to general terms, for each literature search, we included an additional search term for the relevant variable (Table S1b). If any search returned too many results to screen due to resource constraints, only the first 300 search results of each database were screened. This cut-off follows the recommendation of Haddaway et al. (2015) for maximising relevant results from the academic literature in Google Scholar. To check this cut-off, we confirmed in our pilot study that Google Scholar results 300–500 did not contain any eligible studies.

### Study records

We used the systematic review management software Rayyan (Ouzzani et al., 2016) to manage study records, screen abstracts, and track inclusion/exclusion justifications. A folder containing all search results (*FosterMA7_SearchResults*) is available from the OSF project page. Data were extracted from studies, coded, and entered into our data extraction template. A research assistant double-coded study effect estimates in order to identify any coding errors. Discrepancies were resolved by consulting the original text and discussion with the research team. As recommended by Muka et al. (2020), we piloted our data extraction form using the 41 loan repayment studies identified in Gehrig et al. (2021), all of which were included in our final list of eligible studies.

To mitigate inclusion bias and HARK-ing (Hypothesising After the Results are Known) (Kerr, 1998), the primary reviewer (DF) made efforts to avoid learning the results of loan repayment studies that would potentially be included in the meta-analysis, only reading the Methods sections and not the Results sections. All hypotheses were derived a priori from evolutionary theories of cooperation, and without reference to the results of group loan repayment studies.

### Outcomes and prioritisation

Primary outcome variables included all measures of loan repayment efficacy, including measures of default, repayment delinquency, arrears, and loan group survival time. We interpret greater repayment efficacy as groups displaying higher levels of cooperation. This may be intentional on the part of the borrowers, where non-repayment represents an intentional attempt to free-ride on the efforts of other group members. In other cases, borrowers may fail to repay their loan due to bad luck or circumstances outside the borrower’s control, without an explicit intention to free-ride. However, even when non-repayment is not intentional, risky loan investment decisions by borrowers and a lack of sufficient monitoring by their loan group members are themselves plausibly demonstrative of a lack of cooperation in the loan group (Ghatak & Guinnane, 1999). Ultimately, borrowers who fail to repay their share of a group loan prioritise their individual welfare over that of the loan group, so that lower repayment efficacy can generally be considered indicative of non-cooperation within the loan group.

### Risk of bias in individual studies

We were unable to find a tool that suited our need to assess the risk of bias for observational studies in non-clinical settings. We therefore designed a new tool – ROBOS (‘Risk of Bias in Observational Studies’), which requires answering prompts to record factual information about how studies were conducted. This information is used to form judgements of the risk of bias for each study across four domains of potential bias (study design, sampling method, data collection, and analysis methods), and for the study overall. For our primary analysis, we restricted our data estimates to those from studies scoring a ‘Low’ or ‘Moderate’ risk of bias, and conducted sensitivity analyses to test the effects of including results from studies with a ‘High’ risk of bias. ROBOS assessments conducted for all studies are available at https://osf.io/wsdjn.

### Data synthesis

Effect estimates are reported using a variety of statistical measures across different studies and the best way to compare or combine them in a meta-analysis is non-trivial. Our pilot study yielded 14 different effect measures reported across loan repayment studies, the majority of which were log odds. Thus, based on our pilot analyses (see pre-registration and SM1), where we explored multiple ways of comparing and combining different effect measures while maintaining viable sample sizes and interpretability of the meta-analytic estimate, we decided to conduct meta-analyses of log odds outcomes only. An additional advantage of analysing log odds is that its posterior distribution is close to normality, even for relatively small sample sizes (Gelman et al., 2013). To enable comparison between effect estimates from studies with different but comparable outcome variables, we coded all estimates so that positive effect signs reflect increased loan repayment (cooperation), and negative effect signs reflect reduced loan repayment (non-cooperation).

### Meta-analytic model

We expected that many of the eligible studies in our sample would not meet our strict risk of bias threshold, resulting in small sample sizes for our primary analyses. For small sample sizes, Bayesian methods are often preferable due to improved estimation relative to frequentist alternatives (Meager, 2019; Williams et al., 2018) and for avoiding the common problems associated with null hypothesis significance testing (Gelman, 2018). We therefore fit Bayesian multilevel models to estimate meta-analytic associations for each of our 12 variables on group loan repayment. We used a random effects meta-analytic model in which effect sizes are nested in studies, and we assume that the ‘true’ effect of the predictor being modelled differs between studies, either due to actual variation in the effect, or due to sampling variability. The Bayesian meta-analytic model takes the form:

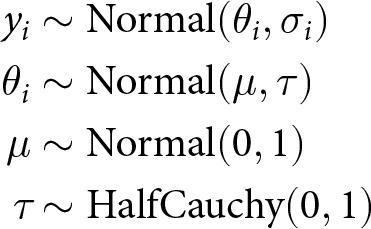
where the effect estimate *y* from study *i* is assumed to be drawn from a Normal distribution with mean *θ_i_* and standard deviation (sd) *σ_i_*. We assume that *θ_i_* is itself drawn from a Normal distribution, where *μ* is the mean of the distribution of effect sizes, and *τ* is the sd around that mean. These two parameters represent the two primary estimands of any meta-analysis: *μ* is the average association across all exchangeable studies, and *τ* is the sd of effects between studies. A further advantage of Bayesian models is the ability to interpret posterior distributions as probability distributions of the effects of interest (Salanti et al., 2019). Our models provide us with posterior distributions for the meta-analytic effect size *μ*, and for the heterogeneity of the underlying effect *τ* (the between-study variance). This allows us to model the uncertainty associated with the meta-analytic effect for each of our predictors of loan repayment, and *τ* additionally allows us to estimate how likely it is that our meta-analytic effects are generalisable to other settings (Vivalt, 2020).

Because individual studies show conflicting results regarding the direction and magnitude of effects (Gehrig et al., 2021), we have little robust information to inform our choice of priors. For our meta-analyses of log odds estimates, we therefore assigned a Normal prior centred on zero for *μ*, to spread the probability diffusely and allow the data to dominate our inferences, as recommended by Gelman (2019). For *τ*, we used a half-Cauchy prior, a distribution with a broad peak around zero and a heavy tail, recommended as a generally applicable prior without strong accompanying assumptions (Gelman, 2006; Polson & Scott, 2012), and given that the between-study variance must be positive (Brockwell & Gordon, 2001). This choice of prior also reduces the risk of inflated estimates for the summary effect *μ* (Williams et al., 2018). We explored the impact of our choice of priors for both *μ* and *τ* via sensitivity analyses (Table S3).

The effect estimates that provide data for the model are weighted using the generic inverse variance method, in which each study’s effect estimate is given a weight equal to the inverse of its variance. This method provides more weight for studies with larger samples and therefore estimates with smaller standard errors, and thus reduces the imprecision of the meta-analytic effect estimate (Higgins & Thomas, 2019). We use the median of the posterior distribution as point estimates, as this parameter value minimises expected absolute loss (McElreath, 2016), and reduces the weight attached to outliers. Generalisability was assessed using between-study variance, *τ*, with higher between-study variance indicating lower generalisability of meta-analytic estimates (Supplementary Materials SM6). Following Spiegelhalter et al. (2004), values of 0.1 < *τ* < 0.5 were interpreted as showing ‘reasonable generalisability’, 0.5 < *τ* < 1 as ‘low generalisability’, and *τ* > 1 as ‘very low generalisability’. Publication bias was assessed using Bayesian Copas selection models (Copas, 1999; Copas & Shi, 2001), which assume that the probability of a study being selected (i.e. published) is a function of the size of its effect estimate and the standard error of this estimate (Supplementary Materials SM8). For each variable, we calculated Bai’s *D* (Bai et al., 2020), for which values close to 0 indicate that posterior distributions of our original model and the equivalent Copas selection model are nearly identical (suggesting negligible publication bias), and values close to 1 indicate non-overlapping distributions (suggesting severe publication bias).

We used R (R CoreTeam, 2020) via RStudio (RStudio Team, 2020) and *brms* (Bürkner, 2017) to fit our Bayesian meta-analytic models in the Stan programming language (Carpenter et al., 2017). Stan enables Markov Chain Monte Carlo sampling with the No U-Turn Sampler (Hoffman & Gelman, 2014; McElreath, 2016) to approximate the posterior distributions of model parameters. The analysis scripts we used are available from: https://github.com/dugaldfoster/MicrofinanceMetaAnalysis.

## Results

### Literature searches

Our 12 separate literature searches returned 3,349 studies. Screening of these studies revealed 225 studies featuring quantitative analysis of the relationship between joint liability loan repayment and at least one of our predictors of interest. From these studies, we extracted 245 relevant effect estimates, as some studies contained multiple predictors of interest. Together with the 85 estimates obtained in our pilot study, this provided a total of 330 estimates (Table 2). In total, 114 of these effect estimates were found to be comparable and 74 of them had a Low/Moderate risk of bias. Table S2 describes literature search results in greater detail.

**Table 2.** The number of relevant effect estimates identified in our pilot study (column ‘Pilot study’), main study (column ‘Main study’), the total number of estimates (column ‘Total’), the number of exchangeable or comparable estimates, i.e. estimates from studies using the same predictor measures, outcome measures, and effect measures (column ‘Comparable’), and the number of comparable studies rated as having a low or moderate risk of bias (column ‘Comparable and low/moderate RoB’)

Variable	Pilot study	Main study	Total	Comparable (% of total)	Comparable and low/moderate RoB (% of total)
VC1. Relatives in the loan group	10	8	18	2 (11)	2 (11)
VC2. Prior acquaintance of group members	18	11	29	8 (28)	6 (21)
VC3. Group tenure	17	32	49	12 (24)	7 (14)
VC4. Frequency of group meetings	5	10	15	2 (13)	0 (0)
VC5. Geographic proximity of group members	11	14	25	7 (28)	5 (20)
VC6. Member management	NA	21	21	12 (57)	7 (33)
VC7. Group sanctions	15	17	32	10 (31)	6 (19)
VC8. Peer monitoring	9	19	28	9 (32)	7 (25)
VC9. External monitoring	NA	16	16	8 (50)	3 (19)
VC10. Group size	NA	31	31	15 (48)	9 (29)
VC11. Borrower age	NA	34	34	13 (38)	11 (32)
VC12. Borrower sex	NA	32	32	16 (50)	11 (34)
**Total**	**85**	**245**	**330**	**114 (35)**	**74 (22)**

### Descriptive statistics

**Figure 1.**
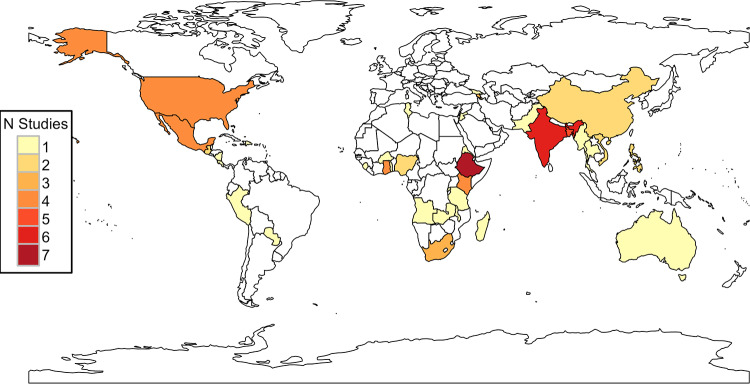
A map of the distribution and frequency of occurrence of the 36 countries from which studies of joint liability loan repayment have been included in our dataset. Many more loan repayment studies exist than are shown here, as our data are restricted to studies that include quantitative analysis of at least 1 of our 12 predictors of interest.

Our dataset of 330 eligible studies from which estimates were drawn spans 36 countries (Figure 1). The main regions represented are North and Latin America, sub-Saharan Africa, and South Asia, reflecting the areas in which group lending is most prevalent. The greatest number of microfinance repayment studies in our dataset are from Ethiopia (seven studies), India (six studies), and Bangladesh (five studies). Moreover, 63% of borrowers across all studies in our dataset were female. The mean age of borrowers was 36.52 years (sd 7.60), and the mean number of borrowers per loan group was 7.16 (sd 4.08). Study sample sizes ranged from 38 to 81,852 borrowers, with a median of 240 (sd 15,701).

### Risk of bias assessments

Our dataset contains estimates from 3 studies rated as having a low risk of bias, 31 studies rated as having a moderate risk of bias, and 14 studies rated as having a high risk of bias. As specified in the study pre-registration, our primary analyses exclude estimates from studies rated as having a high risk of bias. Results of the sensitivity analyses testing the impact of including high-risk-of-bias studies are reported in Table S4.

### Meta-analysis results summary

**Figure 2.**
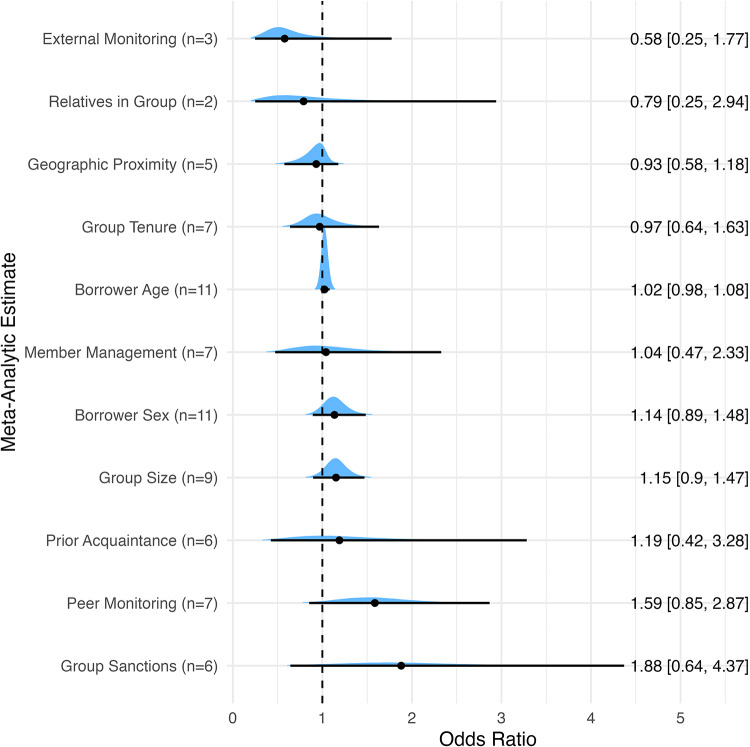
Posterior distributions (blue shading), posterior medians (black dots), and 95% credible intervals (black solid lines) of odds ratios for the meta-analytic association between each variable and group loan repayment, arranged by magnitude of association. If the posterior distribution includes the dotted line representing an odds ratio of 1, then we cannot rule out a null effect. Number of studies for each variable, *n*, is shown in brackets.

We collected sufficient data to meta-analyse the association between 11 variables and loan repayment, with insufficient data for VC4 Frequency of Group Meetings. All models displayed suitable convergence (R-hat values all < 1.01) and stable trace plots (see McElreath, 2016). Our main results are displayed as a forest plot of odds ratio (OR) estimates in Figure 2. Figures S1–S11 show separate forest plots for each variable, showing OR estimates for each study that contributed to that variable’s overall estimate. See Table S4 for sensitivity analyses conducted separately for each variable exploring the impact of (a) using alternative priors, (b) splitting studies by level of analysis (repayment measured at the individual- vs group level), and (c) including studies with a high risk of bias. Our results are generally robust to alternative modelling choices, with minor differences in results when using alternative priors, and results shifting slightly and unsystematically when including high-risk-of-bias studies. For some variables, there is evidence of differences between meta-analytic estimates of studies measuring repayment outcomes at the individual vs the group level, although 95% credible intervals (CI) in all sensitivity analyses include OR of 1 indicating no certain effect. Datasets used in the final analysis are available from the OSF project page (https://osf.io/wsdjn/) and the GitHub page (https://github.com/dugaldfoster/MicrofinanceMetaAnalysis) to facilitate comparison and to provide a resource for future systematic reviews and meta-analyses.

The 95% CI for all 11 meta-analytic estimates include OR of 1 (Figure 2), so we cannot confidently rule out null associations for any variable. We can however use the empirical cumulative distribution function to calculate the probability that any OR estimate is greater or less than 1, indicating a positive or negative association with loan repayment, respectively. In order of probability, the following variables are most likely to have positive associations with group loan repayment: peer monitoring between borrowers (94%), group sanctions (90%), larger groups (90%), female borrowers (87%), and older borrowers (86%). These associations vary widely in their magnitudes, showing between a median 2% increase in the odds of repayment for borrowers who are a year older, and a median 89% increase in the odds of repayment for loan groups that implement sanctions for non-repayment. These estimates also vary widely in their certainty, with 95% CI spanning a difference in OR of 0.1 for the association between borrower age and repayment, and 3.77 for group sanctions. The generally wide CI make it difficult to distinguish clearly between likely negative, null, or positive associations, although the ability to characterise this uncertainty is an advantage of our Bayesian approach.

Two variables are more likely to have negative associations with successful group loan repayment: external monitoring of the group (87%) and closer geographic proximity between members (75%). Magnitudes range from a 7% decrease in the odds of repayment for groups with geographically closer borrowers, and a 42% decrease in the odds of repayment for groups that are externally monitored.

There is a lower probability of positive associations with repayment for acquaintance between borrowers prior to joining a loan group (65%), and management of membership by the group through self-selection and screening (54%). There is also a lower probability of negative associations with repayment for a larger proportion of relatives in a loan group (65%), and longer group tenure (56%). These four variables are likely to have either weak or null associations with loan repayment, although the posterior distributions for some estimates have long tails.

There were an insufficient number of comparable estimates of the association between loan group meeting frequency and loan repayment to meet our criteria for meta-analysis. These studies are characterised by a general lack of consistency in measures of meeting frequency, making it difficult to assess its overall relationship with loan repayment. Studies used variables as diverse as ‘1–5 indicators, with higher value indicating more frequent meetings’ (Bastelaer & Leathers, 2006), ‘group meetings regularly attended’ (Singh & Padhi, 2017), and the treatment effect for a randomised control trial (RCT) that manipulated meeting frequency for a treatment group from fortnightly to monthly, but also required only one representative of each group to participate (Pellegrina & Michele, 2017). Of the five studies identified in the pilot study, all five reported null associations between ‘more regular’ meetings and loan repayment, although the baseline regularity of meetings varies between studies. Of the two studies from the main literature search identified as having comparable predictor, outcome, and effect measures (Berg et al., 2015; Singh & Padhi, 2017), both showed a null association between more regular meetings and loan repayment.

Sensitivity analyses of our priors indicated that our findings are robust to alternative choices of priors for *μ* and *τ* (Table S4). Meta-regressions including various moderators (number of study covariates, group size, borrower age, and borrower sex) failed to show better performance than our original meta-analytic models reported above with no moderators (Table S5). Generalisability estimates using between-study variance yielded mixed results, with 5 of the 11 variables classified as having ‘reasonable generalisability’, 4 as having ‘low generalisability’, and 2 as having ‘very low generalisability’ (Table S6). Finally, there was little indication of publication bias, with no variable’s value of Bai’s *D* exceeding 0.1 (Table S7), although this is perhaps unsurprising given that none of our meta-analyses demonstrated clear evidence of an effect of the variable on loan repayment.

## Discussion

The global popularity of joint liability microfinance loans enabled us to create a cross-cultural dataset on human cooperation in the form of group loan repayment, featuring a range of diverse, non-WEIRD (Henrich et al., 2010) societies. Following Lamba (2014) and Gehrig et al. (2021), we used this dataset to test predictions derived from evolutionary theories of cooperation, given that joint liability microfinance repayment constitutes a cooperative dilemma. We meta-analysed the associations between 11 variables of evolutionary interest and successful loan repayment. To our knowledge, this is the first attempt to quantitatively meta-analyse the microfinance loan repayment literature informed by evolutionary theories of cooperation. We discuss our results grouped by their associated predictions.

### Common ancestry and kin selection

We predicted that loan groups composed of a higher proportion of genetic relatives would exhibit higher repayment rates. There was weak evidence against this prediction from our meta-analysis, which provided a posterior distribution indicating a 65% probability that loan groups with a higher proportion of relatives have lower odds of successfully repaying their loan. This result should be interpreted with caution, as the estimate is uncertain, and the analysis included only 2 studies out of the 18 that we identified in the microfinance literature that include data on the relationship between borrower relatedness and repayment.

Our results suggest repayment in loan groups is not driven by kin-directed cooperation, and that there might even be a negative association between borrower relatedness and repayment. Gehrig et al. (2021) similarly found predominantly negative or null associations between the presence of relatives in loan groups and repayment outcomes. Gehrig et al. (2021) proposed three potential explanations for this: the difficulty of enforcement between relatives due to shared genetic interests (and therefore additional indirect costs of punishment), prioritisation of the needs of additional family members outside the loan group over the interests of the group, and populations with a high density of relatives leading to kin competition that overrides cooperation at the level of the loan group.

A related potential explanation is that punishment between relatives is too costly, not because of indirect fitness costs but due to the likelihood of regular future interaction with related group members when living in kin-structured populations, in which many cooperative behaviours are driven by kin-based reciprocity (Power & Ready, 2019). If punishing a related defaulter in one’s loan group risks damaging a valuable family relationship, the trade-offs to punishment may instead favour inaction, especially if the consequences of their default (e.g. requiring additional repayment contributions) are shared among the loan group. Default in the kin-based loan groups may therefore reflect the prioritisation of reciprocity-based relationships with kin over group loan repayment. This explanation, and its focus on future reciprocated benefits rather than genetic fitness interests, aligns with other studies which have found that across human societies, reciprocity is generally a stronger driver of cooperation than kin selection (Allen-Arave et al., 2008; Jaeggi & Gurven, 2013; Kaplan et al., 1985).

The uncertainty of the estimate for relatedness may also be due to the inconsistent and poorly specified definitions of ‘relatives’ in the microfinance literature. The two studies that we used in our meta-analysis defined relatedness as the ‘percent of group members having a close relative in the group’ (Ahlin & Townsend, 2007, p.F31) and the ‘percentage of group peers who are relatives of the respondent’ (Qinlan & Izumida, 2013, p.334), without specifying exactly who is classified as a relative vs a non-relative or a close vs distant (or non-)relative. Classic research in the evolutionary human sciences has demonstrated that cooperative behaviour differs towards genetically related family members vs non-genetically related family members (Daly & Wilson, 1988) and that cooperation is moderated by the exact degree of genetic relatedness between individuals (Burnstein et al., 1994; Silk, 1980). While some microfinance studies explicitly identify ‘blood relatives’ (Chowdhury, 2016) or record ‘consanguinity’ relationships (Chen et al., 2020), which could be interpreted as implying genetic relatedness, such instances are rare and still rather imprecise. Future microfinance research would benefit from recording the exact degree of genetic relatedness between group members to improve our understanding of how relatedness impacts loan repayment.

### Prior interaction and reciprocity

We predicted that loan groups composed of borrowers with experience or knowledge of one another’s past cooperative behaviour would facilitate reciprocal cooperation and therefore lead to higher repayment rates. Our meta-analytic results provide three independent tests of this prediction.

First, there was weak supportive evidence for reciprocity from a meta-analysis indicating a 65% probability that groups with previously acquainted borrowers have higher odds of successfully repaying their loan. However, the posterior distribution for this estimate peaks around a null association, and the estimate is uncertain. The data for this analysis were unusual in containing a near 50/50 split between studies, suggesting a significant negative association between borrower acquaintance and repayment, and those suggesting a significant positive association, resulting in a high level of between-study heterogeneity. The level of analysis of contributing studies appears to partly explain this heterogeneity (Table S4), with acquaintance between borrowers associated with better individual-level repayment performance (OR 2.71, 95% CI 0.59–9.76), but worse group-level repayment performance (OR 0.66, 95% CI 0.26–1.93). This finding is hard to reconcile, given that group repayment is a function of individual borrower repayment. It could suggest that existing social ties within a group benefit individuals at the expense of the group, for example, by acquainted members showing leniency for repayment deadlines so that group performance is rated poorly due to delinquency, but individual borrowers ultimately repay their loans. As with cooperation between relatives, reciprocal cooperation may be harder to sustain between acquaintances if the expected value of a relationship means the costs of punishment or risks of retaliation are high, so that poor repayment discipline is tolerated (Wydick, 1999). However, CI for both estimates include null associations, and it is also possible that this result is an artefact of sampling variation, or non-random differences between studies using different levels of measurement for loan repayment.

Second, there was neither evidence for nor against reciprocity from a meta-analysis, indicating a 56% probability that groups with longer tenure have lower odds of successfully repaying their loan. The median of the posterior distribution for this estimate lay just below 1, with a long right tail in the distribution. This is most consistent with a null or very small negative association between the length of time a loan group has existed and its repayment performance. A caveat to this analysis is the risk of selection bias in the measurement of group tenure – loan groups that fail early in their existence due to a lack of time for reciprocity-based relationships to form are inherently less likely to be included in studies of loan repayment, thus distorting estimates of the effect of group tenure.

Third, there was some evidence against reciprocity from a meta-analysis indicating a 75% probability that borrowers who live or work in closer geographic proximity have lower odds of successfully repaying their loan, with a mean estimate of 7% lower odds of repayment for each kilometre increase in proximity. This result contradicts theoretical predictions that group members who are better able to monitor one another should display better repayment performance (Besley & Coate, 1995). However, geographic proximity between borrowers may not be an accurate proxy of monitoring propensity, and it may be correlated with other environmental factors that influence repayment (Karlan, 2007). Another possible interpretation is that for individuals to enter a group with geographically distant partners, there must be strong pre-existing levels of trust, leading to higher repayment rates.

Our findings regarding the role of reciprocity in loan groups parallel those of Gehrig et al. (2021), which showed mixed evidence, with 64% of contributing studies showing a negative or non-significant association between prior borrower interaction and loan repayment. A more precise test of the role of reciprocity within loan groups could be achieved with longitudinal data capable of identifying temporal patterns of individual repayment in response to the repayment decisions of fellow group members. If reciprocity between borrowers underlies repayment in group lending, it could be responsible for both higher and lower repayment rates. We would therefore predict individual default in one repayment round to cause default by other group members in the next round, in a tit-for-tat pattern (Axelrod & Hamilton, 1981; Trivers, 1971). In an analysis of group lending data from an Indian lending institution, borrowers were 10–15 percentage points more likely to repay their loans when all other group members repaid (Breza, 2012). However, this effect was asymmetric in that successful peer repayment promoted individual repayment, but peer default was not followed by an increase in individual default.

### Partner choice

We predicted that loan groups formed by borrowers themselves, and those which actively manage their membership through screening, would exhibit higher repayment rates. This prediction was unsupported by a meta-analysis indicating a 54% probability that groups which manage their membership have higher odds of successfully repaying their loan. The posterior distribution for this estimate peaks around a null association. Between-study heterogeneity for this analysis was fairly extreme, which may reflect variation in the choice of measurements of self-formation and screening of new borrowers. The estimate provided by Asgedom et al. (2015) is negative but also highly uncertain, and the only statistically significant negative estimate included is from the only experimental study, in which borrowers participated in a lab-in-the-field microfinance game (Cassar et al., 2007). However, the game played in this study had questionable external validity, given that repayment decisions in the game resulted in returns to borrowers that were kept private. It is the influence of these two studies, which draws the meta-analytic estimate down. Still, our results generally reflect the findings of Gehrig et al. (2021), who found a greater number of studies providing statistically significant positive associations than negative associations between partner choice and group loan repayment.

It is surprising that there is not stronger evidence of a positive association for partner choice, given that borrowers have information on the cooperativeness of potential partners. However, no studies measured ongoing partner choice within the lifetime of the loan group, such as whether a group had ever expelled or replaced a borrower for poor repayment performance. We predict that if partner choice was measured in this way, its association with successful loan repayment would be stronger.

### Punishment

We predicted that loan groups, which carried out sanctions against defaulters, and groups, which conducted peer or external monitoring, would exhibit higher repayment rates. Our results provide three tests of this prediction.

First, this prediction was weakly supported by a meta-analysis indicating a 90% probability that groups which sanction defaulters have higher odds of successfully repaying their loan, with a median estimate of 89% greater odds of repayment. This estimate is uncertain and displays high levels of between-study heterogeneity, although this is mostly driven by variation among positive estimates from the original studies. While this suggests a positive overall association between punishment and repayment, all studies contributing to our analysis used measures of potential punishment, rather than measuring instances in which punishment for non-repayment of loans actually occurred. This proxy measure is common in studies of human punishment, although the assumption that the threat of punishment functions to convert would-be free-riders into cooperators is debatable (Raihani & Bshary, 2019). Evidence suggests instead that punishment is motivated by inequity aversion, whereby individuals are sensitive to differential outcomes between themselves and their interaction partners (Fehr & Schmidt, 1999; Raihani & McAuliffe, 2012) and use punishment to alter their relative pay-offs. This is supported by an international analysis of microfinance repayment, which concludes that higher levels of inequity aversion among borrowers predict increased default rates (Jordan et al., 2019), as groups refuse to repay the dues of members who are perceived to have profited unfairly through strategic default.

As noted in the Introduction section, little research has examined actual mechanisms of punishment within joint liability loan groups, beyond anecdotal evidence of social shaming, confrontation and confiscation of possessions (Solli et al., 2015). The group-based nature of such punishment behaviour provides support for theories that focus on the sharing of punishment costs (Boyd et al., 2010, 2003). Future research should explore the relative costs and benefits to individual members involved in microfinance punishment scenarios, as punishment can only evolve as a mechanism if there is a correlation between punishment behaviour and the benefits of cooperation which the punisher receives (Gardner & West, 2004).

Second, this prediction was supported by a meta-analysis indicating a 94% probability that groups which conduct peer monitoring have higher odds of successfully repaying their loan. This estimate is still fairly uncertain, although more certain than the estimate for group sanctions. Some of this uncertainty may be due to variation in the variables used as proxy measures of monitoring, including the regularity of visits between members, and knowledge of other group members’ business activities. Peer monitoring is one of the main functions of the loan group and the majority of peer monitoring may occur discreetly during dedicated events, such as repayment meetings in which members report their investment returns.

Third, there was some evidence against this prediction from a meta-analysis indicating an 87% probability that groups that undergo external monitoring have lower odds of successfully repaying their loan. This estimate is more certain, although the posterior distribution has a long right tail. While it may seem counterintuitive that externally monitored groups show worse repayment rates, this finding may be due to reverse causality. Most MFIs do not have the resources to conduct monitoring of groups, reserving inspection visits for groups with historical repayment problems (Armendáriz & Morduch, 2010).

### Group size, borrower age, and borrower sex

Due to their regular inclusion as covariates in studies of loan repayment, the meta-analyses of group size, borrower age, and borrower sex had the largest sample sizes. Hence, their estimates showed the greatest certainty. Our analysis provides some evidence that larger groups are associated with improved repayment performance, contradicting theoretical predictions that larger groups should make the establishment of reciprocal relationships more difficult (Boyd & Richerson, 1988). However, this association between group size and cooperation is unlikely to be linear, and may only apply within a certain range: group sizes in our dataset ranged from 2 to 18, which may reflect upper limits on borrowers’ ability to track cooperative behaviours.

Borrower age provided the most certain estimate of all our analyses, although the magnitude of the association is close to null, likely because the association represents the difference in repayment from only a single year increase in age. There is stronger evidence of a positive association between repayment and being female, which supports the common assumption that women generally show better repayment performance (Armendáriz & Morduch, 2010), possibly because they tend to be more risk-averse in their loan investment choices or more responsive to the threat of social shaming. The 63% female makeup of our dataset reflects a historical focus on women in microfinance, particularly in joint liability lending (D’espallier et al., 2013) and is consistent with a global analysis of 350 lending institutions, which found 73% of borrowers were female (D’espallier et al., 2011).

### Limitations

Several limitations necessitate caution in the interpretation of our results, especially for those considering their practical application in a novel lending context. Restricting our sample to comparable effect estimates – a prerequisite for any meta-analysis – reduced our meta-analytic sample sizes, so that for each variable we were only able to analyse between 11% and 57% of estimates available in the literature. This was further exaggerated in our subgroup and sensitivity analyses. All of our meta-analyses constitute univariate analyses as we were unable to conduct multivariate meta-regressions capable of controlling for known confounds (Table S5), largely due to the lack of shared covariate sets between studies.

Our study findings may also be affected by endogeneity in loan group formation, whereby economic or environmental conditions cause groups to form in a certain way (Berhane et al., 2009). For example, borrowers may be more likely to form groups with family members during an economic downturn. Assuming such an economic downturn would also increase default rates due to poor business performance, this could result in a correlation between borrower relatedness and default, even though, in a counterfactual scenario, borrowers may have had even higher default rates had they not formed groups with family members. Alternatively, some variables such as group size may not be exogenous to the maintenance of cooperation. Large groups may be large precisely because some other mechanism is already in place that facilitates cooperation, causing more people to join that group. This endogeneity may explain the theoretically unexpected finding that larger groups are associated with higher repayment. Additionally, there is a risk of site selection bias among studies of loan repayment, if lending institutions that collaborate with researchers differ in some non-random way from institutions that are not engaged in research, such as having better repayment performance (Allcott, 2015; Reid et al., 2018).

Our eligible studies display considerable variation in study settings, including variation in features of joint liability loan contracts that could influence repayment decisions. For example, interest rates vary within and between lending institutions, and higher or lower rates could influence the costs and benefits of loan repayment for borrowers. Loan features such as interest rates and loan size could also correlate with higher-order factors associated with loan repayment outcomes, such as the capacity or experience of the lending institution, or adverse selection of riskier borrowers (Aleem, 1990; Bottomley, 1975). We could not control for such factors in our analyses.

There is variation in the measurement of predictor and outcome variables, even among comparable studies. When combining predictor variables, we aimed to strike a balance between identifying functionally similar variables and maximising the sample sizes available for our analyses, but due to differences in expert judgement there are likely to be reasonable objections to some of our categorisation decisions. To improve the generalisability of mechanisms promoting cooperation and loan repayment, we encourage broader collaboration between microfinance researchers, particularly joint efforts to collect data on the same variables for loan groups borrowing from different lending institutions, and to test the same theoretical models of repayment in different study contexts (Vivalt, 2020).

Loan repayment studies which measure only whether loans were ultimately repaid at the group level may be overlooking the most interesting data on cooperation between borrowers: the proportion of successfully repaid group loans that involved any instance of individual default. Groups that face individual default but which successfully overcome this through fully repaying their loan have arguably engaged in a more costly act of cooperation than groups in which all borrowers repay their dues. Conversely, studies which measure only individual repayment performance cannot assess whether the cooperative dilemma of group lending was solved. While some studies do report individual- and group-level delinquency separately (Bassem, 2008), no studies report the combined results. This mirrors a measurement problem among lending institutions, which tend to measure loan repayment only at the institutional level (Rosenberg, 1999), reflecting their lack of concern regarding *how* group loans are repaid – only *that* they are repaid.

Finally, almost all of our estimates are drawn from cross-sectional studies, limiting our ability to infer causal relationships between our predictors of interest and loan repayment. While our meta-analytic estimates provide indirect evidence of causal relationships, and have value in predicting loan repayment outcomes, they should not be interpreted as causal effects. Our understanding of the causal factors that promote cooperation and repayment in group loans would benefit from more studies using randomisation-based designs in natural settings, such as field-based RCTs, enabling manipulation of variables of interest between different treatment groups. There are notable examples of high-quality RCTs in microfinance research (Field et al., 2012; Giné & Karlan, 2014), but these tend to focus on the effects of manipulating features of the loan contract, such as liability structure, rather than manipulating features of joint liability groups likely to facilitate or inhibit cooperation amongst their members.

## Conclusion

Our meta-analyses do not provide conclusive evidence regarding the role of evolutionary mechanisms of cooperation supporting loan repayment in joint liability groups. We find little support for kin selection, reciprocity, and partner choice. The two variables showing the strongest evidence of a positive association with loan repayment are both related to punishment. The predominance of uncertain associations in our results may be due to statistical variation between studies due to imprecise estimates or may reflect true variation in the role of evolutionary mechanisms across different cultural and ecological contexts.

## Supporting information

10.1017/ehs.2026.10047.sm001 Foster et al. supplementary material Foster et al. supplementary material

## Supplementary material

The supplementary material for this article can be found at https://doi.org/10.1017/ehs.2026.10047.

## Research transparency and reproducibility interest

All data and code are available at https://github.com/dugaldfoster/MicrofinanceMetaAnalysis and https://osf.io/wsdjn/.
