# Supplementary material for: Testing evolutionary theories of human cooperation via meta-analysis of microfinance repayment
Source: Evol Hum Sci. 2026 Apr 6;8:e15. doi: 10.1017/ehs.2026.10047 (PMC13122405; doi:10.1017/ehs.2026.10047)
Supplement: Foster et al. supplementary material [file S2513843X26100474sup001.pdf]

Supplementary Materials for

“Testing Evolutionary Theories of Human Cooperation via Meta-Analysis of Microfinance Repayment”

Dugald Foster, Erik Postma, Shakti Lamba, Alex Mesoudi*

Centre for Ecology and Conservation, University of Exeter Cornwall Campus, Penryn, TR10 9FE, United Kingdom.

*Corresponding author: Alex Mesoudi, a.mesoudi@exeter.ac.uk

Contents

SM1. Pilot Study....3
Model Selection Criteria....4
Risk of Bias Assessment....4
SM2. Literature Search....6
Search Terms....6
Table S1a. Primary search terms....6
Table S1b. Additional search terms for individual searches....6
Search Results....7
Table S2. Literature search results for each database used in each meta-analysis...7
SM3. Prior Sensitivity Analysis....10
Table S3. The priors tested for the parameters in our meta-analytic models....10
SM4. Investigating Between-Study Heterogeneity....11
Table S4. Sensitivity analysis results....11
SM5. Investigating Meta-Analytic Effect Moderators....13
Table S5. Model comparison results for effect moderators....14
SM6. Generalisability Estimates....15
Table S6. Estimates of between-study heterogeneity for each variable....15
SM7. Supplementary Figures S1-S11....16
Figure S1. Forest plot for variable VC1 Relatives in Group....16
Figure S2. Forest plot for variable VC2 Prior Acquaintance....17
Figure S3. Forest plot for variable VC3 Group Tenure....18
Figure S4. Forest plot for variable VC5 Geographic Proximity....19
Figure S5. Forest plot for variable VC6 Member Management....20
Figure S6. Forest plot for variable VC7 Group Sanctions....21
Figure S7. Forest plot for variable VC8 Peer Monitoring....22
Figure S8. Forest plot for variable VC9 External Monitoring....23
Figure S9. Forest plot for variable VC10 Group Size....24
Figure S10. Forest plot for variable VC11 Borrower Age....25
Figure S11. Forest plot for variable VC12 Borrower Sex....26
SM8. Publication Bias Analysis....27
Table S7. Bai's D measure of publication bias for each variable....27
Supplementary Materials References....28

SM1. Pilot Study

In planning our analyses, we faced a number of methodological obstacles that were difficult to resolve in abstract. We therefore conducted a pilot study using the 41 studies identified in the most recent review of the group loan repayment literature (Gehrig et al., 2021). Our pilot dataset included results from 189 statistical models, analysing the relationship between 663 unique predictors and 65 repayment outcomes, using data collected on borrowers from 36 microfinance institutions based in 59 regions across 32 countries (the full pilot dataset *FosterMA8_DataExtracted_Pilot_Split* is available on the OSF project page). The dataset showed a wide range of variable types analysed in loan repayment studies (examples are displayed in Table SM1 of Gehrig et al., 2021), including binary, categorical and continuous measures of predictor variables, measured at the borrower- and loan group-level. Meta-analysis is technically possible with just two estimates, although an analysis of all meta-analyses contained in the Cochrane Database of Systematic Reviews identified a median of three studies per meta-analysis (Davey et al., 2011). We therefore decided to conduct separate meta-analyses for each measure of effect for which at least three estimates were provided, assuming contributing studies were sufficiently homogenous in their study design and measurement of variables. In our subsequent pilot analysis of 6 variable categories, this almost always meant conducting meta-analyses of log odds ratios, which were the most frequently reported effect measure in our pilot dataset.

We considered whether we should meta-analyse all variable categories associated with a mechanism together (e.g. producing one meta-analytic estimate for the effect of “Prior Interaction” on loan repayment, combining estimates from all four of the associated variable categories), or whether we should analyse each variable category separately. We decided on the latter for several reasons. First, we found extensive variation in the methods used to measure the same variables between studies, and the statistical methods used to estimate their impact on loan repayment. We decided to minimise this measurement and statistical heterogeneity by focussing on synthesising results for individual variable categories. Second, evolutionary mechanisms have not been directly assessed in loan repayment studies, as the literature on the evolution of cooperation and the literature on microfinance loan repayment have developed independently until now. This means we needed to find suitable proxies of evolutionary mechanisms in studies from the microfinance literature. We decided it would be more useful for the field of microfinance research to estimate meta-analytic effects for variables which are commonly studied in this literature. Third, the more variable categories are associated with a mechanism, the harder it would be to interpret any meta-analytic effects, especially when the mechanism is associated with multiple, diverse variables (e.g. Prior Interaction). Fourth, by conducting separate meta-analyses for each variable category, those who disagree with us concerning the mechanisms to which they have been assigned are free to interpret them individually. Despite the fact that we assign each variable category to one mechanism, there is likely to be some overlap between mechanisms for multiple variable categories. For example, while geographical proximity reasonably constitutes a proxy for prior interaction, it is also likely that borrowers who live closer to one another are more easily able to deliver punishments for defaulters. Similarly, while relatedness between borrowers denotes common ancestry and could therefore promote kin-directed cooperation, it is also likely that relatives have more experience

of interacting with one another, enabling the formation of cooperative relationships based on reciprocity.

Model Selection Criteria

We decided to extract one estimate per study for each meta-analysis we conduct, under the reasoning that one estimate consistently selected from each study is preferable to multiple estimates from differing model specifications, even if this comes at the cost of a smaller total sample of effect estimates. By choosing one estimate per study we also avoided the statistical and inferential issues associated with three-level meta-analysis: extracting multiple estimates from the same studies would introduce statistical dependencies between effect estimates, invalidating one of the core assumptions of conventional meta-analysis (Cheung, 2014; Van den Noortgate et al., 2013). For each study, we used the following criteria to select a model from which to extract effect estimates (NB the criteria are hierarchical, so that competing models are judged on the basis of how well they meet the criteria in the order they appear.):

1. The model is the only model in the study
2. The model includes the most relevant predictor variable
3. The model is the most statistically appropriate for the type of data being analysed
4. The model includes the most relevant outcome variable for loan repayment
5. The model provides the most comparable effect measure (e.g. log odds)
6. The model is applied to the largest sample of data
7. The model includes our two pre-specified controls (a measure of borrower SES, wealth, or education, and a measure of loan size or interest rate), with the fewest additional covariates
8. The model is the best-fitting model

We selected models from studies independently for each variable category, so that different models from the same study could potentially provide estimates for different meta-analyses. Estimates from variables included in models as controls are considered unreliable (Westreich and Greenland, 2013) and ideally should not contribute to meta-analyses. However, based on our pilot study we anticipated severe sample size restrictions using that approach, and we therefore included estimates for variables originally modelled as controls.

Risk of Bias Assessment

Although multiple tools have been developed for assessing the risk of bias of studies in the evidence synthesis literature, during our pilot study we were unable to find a tool that suited our need to assess primarily cross-sectional studies in non-clinical settings. We therefore designed a new tool which we called ROBOS ("Risk of Bias in Observational Studies"). To do this we took inspiration from the ROBINS-I tool (Sterne et al., 2016), which involves answering prompts to record factual information about how studies were conducted. This information is used to formulate subjective judgements of the risk of bias for each study across 7 domains of potential bias, and for the study overall. We created a simplified, one-page version of the ROBINS-I tool which consolidates the 7 original domains into 4 to be assessed for bias: Study Design, Sampling Method, Data Collection, and Analysis Methods. The ROBOS tool provides a free-text

box to enter information for each domain when assessing a study, with specific criteria to guide the assignment of a “Low”, “Moderate”, or “High” risk of bias rating. Based on this information, we assign an overall risk of bias rating for the study, which takes the value of the highest rating out of all 4 domains. For example, a study which scored “Moderate”, “Low”, “Moderate”, and “High” across the 4 domains would receive an overall rating of “High” risk of bias. While this approach may seem conservative, it follows the approach used in the ROBINS-I tool, and ensures a stricter and more objective distinction between studies at different risk of bias. For our primary analysis we restricted effect estimates to those from studies scoring a “Low” or “Moderate” risk of bias, although sensitivity analyses tested the effects of including results from studies with a “High” risk of bias. Results of our assessments, and a description of how to use the tool, are available in the *FosterMA10_ROBOS* folder on the OSF project page.

SM2. Literature Search

Search Terms

Table S1a. Primary search terms

To be indexed, studies needed to mention at least one of the terms listed in each column (e.g. microfinance AND group loan AND repayment). Asterisks enable searches for all words with the specified root (e.g. a search for “repay*” will return “repay”, “repayment” etc.)

Database	Search syntax (All Fields)
Google Scholar	(microfinance OR microcredit OR microloan) AND ("joint liability" OR "group loan" OR "group lending" OR "solidarity loan" OR "solidarity lending") AND ("repayment" OR "default" OR "delinquent" OR "delinquency" OR "arrears")
Web of Science	(microfinance OR microcredit OR microloan) AND ("joint liability" OR "group loan*" OR "group lend*" OR "solidarity loan*" OR "solidarity lend*") AND ("repay*" OR "default" OR "delinquen*" OR "arrear*")
ProQuest Dissertations & Theses Global	(microfinance OR microcredit OR microloan) AND ("joint liability" OR "group loan*" OR "group lend*" OR "solidarity loan*" OR "solidarity lend*") AND ("repay*" OR "default" OR "delinquen*" OR "arrear*")
EconLit	(microfinance OR microcredit OR microloan) AND ("joint liability" OR "group loan*" OR "group lend*" OR "solidarity loan*" OR "solidarity lend*") AND ("repay*" OR "default" OR "delinquen*" OR "arrear*")

Table S1b. Additional search terms for individual searches.

Variable Category	Additional Search Terms
Relatives in the loan group	AND (“family” OR “kin” OR “relat*”)
Prior acquaintance of group members	AND (“friend*” OR “acquaint*” OR “social tie*”)
Group tenure	AND (“group tenure” OR “group age” OR “loan cycle”)
Frequency of group meetings	AND (“meeting frequen*” OR “frequency of meetings”)
Geographic proximity of group members	AND (“proximity” OR “distance” OR “neighbo*” OR "same village")
Management of group membership	AND (“screen*” OR “group formation” OR “invit*” OR “reject*”)
Group sanctions	AND (“sanction*” OR “punish*” OR “pressure”)
Peer monitoring	AND (“peer monitoring” OR “monitor*” OR “visit” OR “collect information”)
External monitoring	AND (“loan officer” OR “by the MFI” OR “external monitor*”)
Group size	AND (“group size” OR “number of borrowers” OR “number of

	members”)
Borrower Age	AND (“borrower age” OR “average age” OR “young*” OR “old*”)
Borrower Sex	AND (“sex” OR “gender”)

Search Results

Table S2. Literature search results for each database used in each meta-analysis.

Variable Category	Database	N Results	Total Results	Deduplicated Total	Total after excluding non-reviewed papers
VC1. Relatives in the loan group	Google Scholar	10500	482	419	367
	Web of Science	26			
	ProQuest	139			
	EconLit	17			
VC2. Prior acquaintance of group members	Google Scholar	1880	350	322	270
	Web of Science	15			
	ProQuest	28			
	EconLit	7			
VC3. Group tenure	Google Scholar	1220	339	324	257
	Web of Science	2			
	ProQuest	36			
	EconLit	1			
VC4. Frequency of group meetings	Google Scholar	165	174	168	129
	Web of Science	1			
	ProQuest	7			
	EconLit	1			
VC5. Geographic proximity of group members	Google Scholar	5750	326	312	272
	Web of Science	5			
	ProQuest	20			
	EconLit	1			

VC6. Management of group membership	Google Scholar	5360	356	325	289
	Web of Science	10			
	ProQuest	43			
	EconLit	3			
VC7. Group sanctions	Google Scholar	8260	376	332	282
	Web of Science	20			
	ProQuest	44			
	EconLit	12			
VC8. Peer monitoring	Google Scholar	7350	390	350	320
	Web of Science	18			
	ProQuest	64			
	EconLit	8			
VC9. External monitoring	Google Scholar	2770	360	337	302
	Web of Science	1			
	ProQuest	52			
	EconLit	7			
VC10. Group size	Google Scholar	3760	384	354	314
	Web of Science	8			
	ProQuest	71			
	EconLit	5			
VC11. Borrower Age	Google Scholar	8720	337	327	286
	Web of Science	4			
	ProQuest	18			
	EconLit	15			
VC12. Borrower Sex	Google Scholar	9120	364	329	282
	Web of Science	9			
	ProQuest	50			

	EconLit	5			
					**Total**: 3349

SM3. Prior Sensitivity Analysis
To test the influence of our choice of priors, we repeated each analysis while varying our choice of prior for μ and τ, both independently and in combination (**Table S3**). For the μ prior we investigated the impact of reducing the probability of larger meta-analytic effect estimates by halving the original scale parameter, reflecting the reasonable belief that there is a higher probability that any of our estimates is closer to 0, and downweighting more extreme values. This enables us to compare our original results with those produced when using a more conservative prior. We do not test the impact of varying the mean of the prior distribution for μ due to generally insufficient information regarding likely magnitudes of effects across the eleven variable categories we analyse.

Given the small number of studies included in most of our analyses, the lack of information provided by the data regarding levels of between-study heterogeneity could justify the use of a more informative prior for τ (Williams et al., 2018). We therefore investigated the impact of reducing the probability of smaller values by increasing the τ prior mean to 0.5, reflecting the reasonable belief that between-study heterogeneity is not only necessarily positive but likely to be substantial. We follow the advice of Smith et al. (1995) who suggest around a one order magnitude spread of odds ratios between studies is a plausible estimate, which is reached at τ = 0.59. At τ = 0.50, the 97.5 percentile of the distribution of odds ratios is 7.1 times the 2.5 percentile, which is slightly under one order of magnitude. At the same value of τ, the median ratio of the maximum to minimum odds ratio for a randomly selected pair of studies contributing to a meta-analysis is equal to exp(1.09*0.5) = 1.72 (Spiegelhalter et al., 2004). This is equivalent to one study of the pair providing an odds ratio of 1 (no association), and the other study providing an odds ratio of 1.72 (indicating 72% greater odds of loan repayment). Results of our prior sensitivity analyses are shown in **Table S4**.

Table S3. The priors tested for the parameters in our meta-analytic models.

	**Primary Analysis**	**μ Prior Test 1**	**τ Prior Test**	**Combined Test**
**μ**	Normal(0, 1)	Normal(0, 0.5)	Normal(0, 1)	Normal(0, 0.5)
**τ**	HalfCauchy(0, 1)	HalfCauchy(0, 1)	HalfCauchy(0.5, 1)	HalfCauchy(0.5, 1)

SM4. Investigating Between-Study Heterogeneity

Differences between individual- and group-level estimates are reported in **Table S4**. There were no consistent patterns in estimates of heterogeneity between individual- and group-level studies, and the inclusion of studies at a high risk of bias had mixed effects on the original meta-analytic estimates.

Table S4. Sensitivity analysis results.

The table shows updated estimates of odds ratio posterior ~~medians~~means and 95% credible intervals. We explored the influence of: alternative priors, restricting estimates to studies reporting individual- versus group-level repayment, and including estimates from studies rated as having a high risk of bias.

	**Original estimate**	**μ prior check**	**τ prior check**	**Combined prior check**	**Individual level repayment**	**Group level repayment**	**High Risk of Bias**
**Relatives in the Loan Group**	0.81 (0.25 - 2.94)	0.89 (0.40 - 2.02)	0.83 (0.24 - 3.19)	0.90 (0.40 - 2.12)	1.13 (0.21 - 5.95)	0.73 (0.19 - 3.32)	No studies available
**Prior Acquaintance**	1.19 (0.42 - 3.28)	1.11 (0.53 - 2.29)	1.19 (0.42 - 3.33)	1.11 (0.53 - 2.30)	2.71 (0.60 - 8.71)	0.66 (0.26 - 1.93)	1.24 (0.50 - 3.35)
**Group Tenure**	0.98 (0.64 - 1.64)	0.98 (0.67 - 1.53)	0.99 (0.63 - 1.68)	0.99 (0.66 - 1.58)	1.14 (0.66 - 2.12)	0.84 (0.36 - 2.20)	1.18 (0.51 - 2.53)
**Geographic Proximity**	0.90 (0.57 - 1.18)	0.91 (0.63 - 1.17)	0.89 (0.54 - 1.21)	0.90 (0.60 - 1.19)	0.96 (0.66 - 1.25)	0.90 (0.57 - 1.18)	0.92 (0.63 - 1.20)
**Member Management**	1.05 (0.40 - 2.69)	1.03 (0.51 - 2.07)	1.05 (0.40 - 2.73)	1.03 (0.50 - 2.08)	1.06 (0.29 - 3.93)	1.04 (0.40 - 2.69)	0.79 (0.35 - 1.77)
**Group Sanctions**	1.83 (0.63 - 4.40)	1.49 (0.66 - 2.91)	1.81 (0.62 - 4.51)	1.46 (0.64 - 2.89)	2.23 (0.60 - 6.22)	1.83 (0.63 - 4.40)	1.80 (0.73 - 4.18)
**Peer Monitoring**	1.58 (0.85 - 2.87)	1.44 (0.83 - 2.37)	1.58 (0.83 - 3.00)	1.43 (0.80 - 2.40)	1.74 (0.80 - 3.48)	1.58 (0.85 - 2.87)	1.69 (0.29 - 9.93)
**External Monitoring**	0.60 (0.25 - 1.82)	0.71 (0.37 - 1.59)	0.61 (0.25 - 1.86)	0.72 (0.36 - 1.65)	0.55 (0.19 - 2.42)	0.60 (0.25 - 1.82)	0.99 (0.36 - 3.19)
**Group Size**	1.15 (0.90 - 1.47)	1.14 (0.90 - 1.45)	1.15 (0.89 - 1.49)	1.14 (0.89 - 1.46)	1.22 (0.71 - 1.91)	1.15 (0.89 - 1.48)	1.04 (0.85 - 1.25)

**Borrower Age**	1.03 (0.98 - 1.08)	1.03 (0.98 - 1.08)	1.03 (0.98 - 1.08)	1.03 (0.98 - 1.08)	1.03 (0.97 - 1.10)	1.03 (0.98 - 1.08)	1.02 (0.99 - 1.05)
**Borrower Sex**	1.14 (0.89 - 1.49)	1.13 (0.89 - 1.45)	1.14 (0.88 - 1.51)	1.13 (0.88 - 1.51)	1.16 (0.82 - 1.73)	1.14 (0.89 - 1.49)	1.08 (0.91 - 1.33)

SM5. Investigating Meta-Analytic Effect Moderators

Meta-regression can reveal relationships among variables shared between studies contributing to a meta-analysis. It is incorrect to infer causal connections from a meta-regression, as the meta-regression itself is a form of observational study. Even so, meta-regression is useful for generating hypotheses regarding relationships among variables, which can then be studied in future research. We used meta-regression to explore the roles of the following moderator variables. To assess the impact of effect moderators we used approximate leave-one-out cross validation (LOO-CV) (Vehtari et al., 2017) to compare the out-of-sample predictive accuracy for models with and without terms for moderators.

Meta-regression 1: Number of Study Covariates

During our pilot study we learned of significant variation in the number and nature of covariates for estimates from different studies. Our model selection criteria aim to reduce this variation by selecting the model from each study with the fewest covariates in addition to our pre-specified controls, with the aim of including at least partially exchangeable effect estimates (Higgins et al., 2009). Here ~~W~~we ~~also~~ ran a meta-regression to investigate the number of covariates in the models from which effect estimates have been drawn as an effect moderator.

Meta-regression 2: Group Size

While many loan repayment studies do not analyse the effects of loan group size on repayment, they do provide descriptive statistics including the average size of loan groups. Using this information we ran a meta-regression including a term for group size to investigate the role of group size in moderating the effects of each of our variable categories on loan repayment.

Meta-regression 3: Borrower Age

As with group size, many loan repayment studies may not produce direct effect estimates for borrower age, but do report statistics on the average age of borrowers in the population. Using this information we ran a meta-regression including a term for average borrower age, to investigate the role of borrower age in moderating the effects of each of our variable categories on loan repayment.

Meta-regression 4: Borrower Sex

Using the same information contained in loan repayment studies descriptive statistics, we ran a meta-regression including a term for the proportion of males in the study population, to investigate the role of borrower sex in moderating the effects of each of our variable categories on loan repayment.

Model comparison results for meta-analytic models including effect moderators are reported in **Table S5**. No models showed better performance than the original meta-analytic models. We were unable to conduct any meta-regressions including borrower age due to data inconsistencies between contributing studies.

Table S5. Model comparison results for effect moderators

Model comparison results (shown as differences in expected log pointwise predictive density using Bayesian leave-one-out cross-validation, with the standard error of component-wise differences in brackets) between the original model and a model including a term for a) model complexity, b) loan group size, c) borrower age, ~~and~~or d) borrower sex. Models with different likelihoods due to missing covariates cannot be compared using this method - these are marked with "NA".

	**Study Covariates**	**Group Size**	**Borrower Age**	**Borrower Sex**
**Relatives in the Loan Group**	-0.6 (0.2)	-0.6 (0.0)	NA	NA
**Prior Acquaintance**	-1.2 (0.7)	-0.4 (2.2)	NA	NA
**Group Tenure**	-0.2 (0.4)	NA	NA	NA
**Geographic Proximity**	-0.3 (0.5)	-0.3 (0.8)	NA	NA
**Member Management**	-0.2 (0.6)	-0.4 (0.5)	NA	NA
**Group Sanctions**	-0.6 (0.8)	NA	NA	NA
**Peer Monitoring**	-0.2 (0.6)	NA	NA	-0.6 (0.4)
**External Monitoring**	-0.4 (0.8)	NA	NA	-1.2 (0.7)
**Group Size**	-0.3 (0.8)	NA	NA	NA
**Borrower Age**	-0.1 (0.1)	NA	NA	NA
**Borrower Sex**	-1.8 (1.2)	NA	NA	NA

SM6. Generalisability Estimates

Estimates forof τ for each variable meta-analysed are displayed in **Table S6**. We interpret τ in line with the broad categorisation suggested by (Spiegelhalter et al., (2004), whereby values of $0.1 < \tau < 0.5$ are considered as “reasonable” levels of heterogeneity, $0.5 < \tau < 1$ as “fairly high”, and $\tau > 1$ as “fairly extreme”. Reframing these as generalisability estimates, we can consider values of $0.1 < \tau < 0.5$ as showing “reasonable generalisability”, $0.5 < \tau < 1$ as “low generalisability”, and $\tau > 1$ as “very low generalisability”. These classifications for each variable are shown in Table S6: five of the 11 variables are classified as having “reasonable generalisability”, four as having “low generalisability”, and two as having “very low generalisability”. Note however that these classifications are based on the mean estimate of τ. Only one variable (Borrower Age) had 95% credible intervals that did not exceed the “low generalisability” threshold of 0.5; all other variables’ 95% credible intervals included either the “low” or “very low” categories.

Table S6. Estimates of between-study heterogeneity for each variable.

Variable Category	τ (95% Credible Interval)	Generalisability
Borrower Relatedness	0.77 (0.02 - 2.98)	Low
Prior Acquaintance	1.15 (0.18 - 2.69)	Very low
Group Tenure	0.46 (0.06 - 1.14)	Reasonable
Geographic Proximity	0.26 (0.02 - 0.87)	Reasonable
Member Management	1.12 (0.44 - 2.36)	Very low
Group Sanctions	0.96 (0.07 - 2.56)	Low
Peer Monitoring	0.56 (0.04 - 1.42)	Low
External Monitoring	0.81 (0.10 - 2.39)	Low
Group Size	0.24 (0.07 - 0.57)	Reasonable
Borrower Age	0.06 (0.02 - 0.14)	Reasonable
Borrower Sex	0.22 (0.01 - 0.61)	Reasonable

SM7. Supplementary Figures S1-S11

The following forest plots show, separately for each of the 11 analysed variables, the posterior distributions (blue shading), posterior medians (black dots), 95% credible intervals (black solid lines) and original study estimates (unfilled dots) of odds ratios for the meta-analytic association in each study between the variable of interest and group loan repayment, along with the overall posterior distribution and estimates as shown in Figure 2.

Figure S1. Forest plot for variable VC1 Relatives in Group

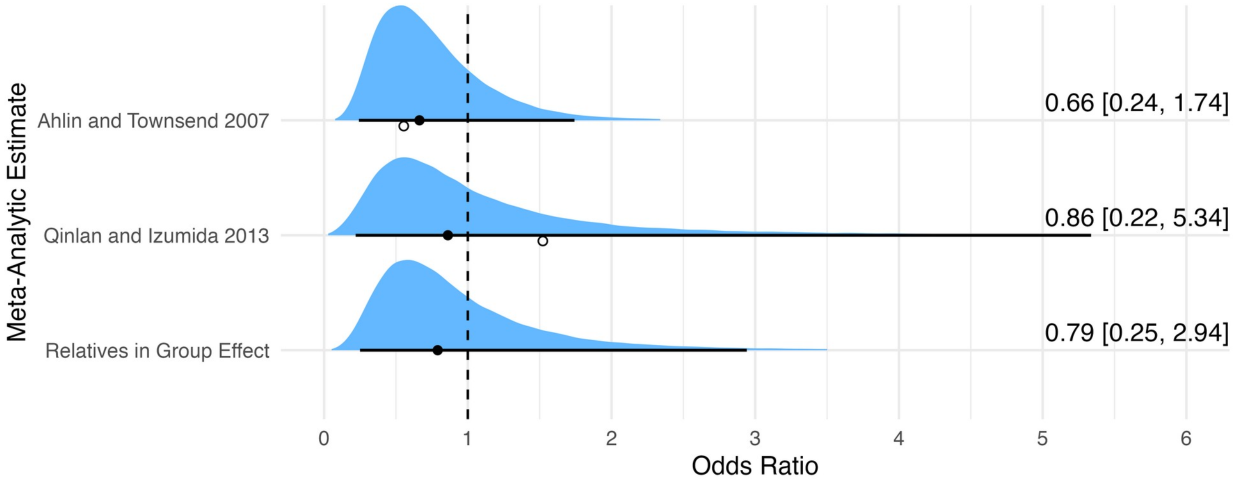

Figure S2. Forest plot for variable VC2 Prior Acquaintance

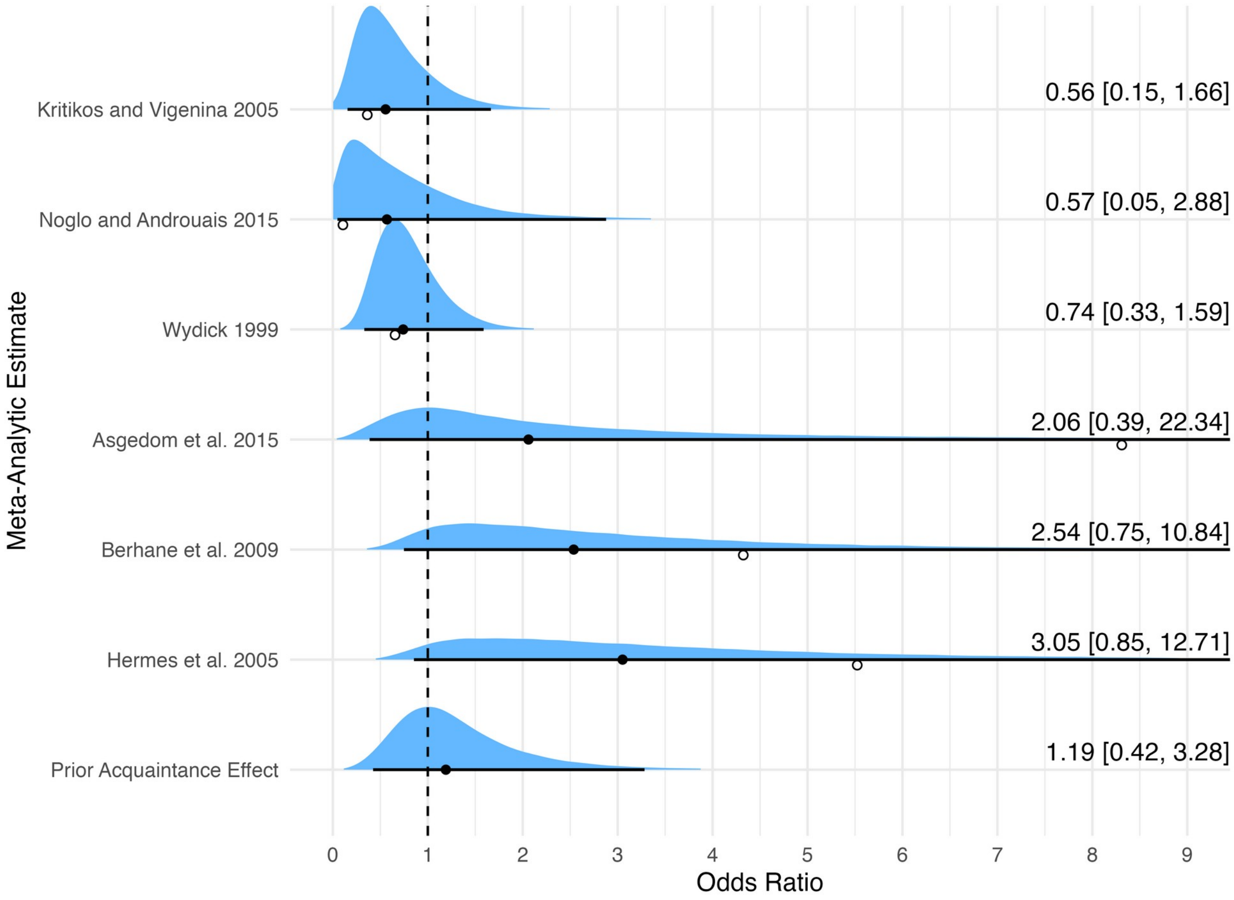

Figure S3. Forest plot for variable VC3 Group Tenure

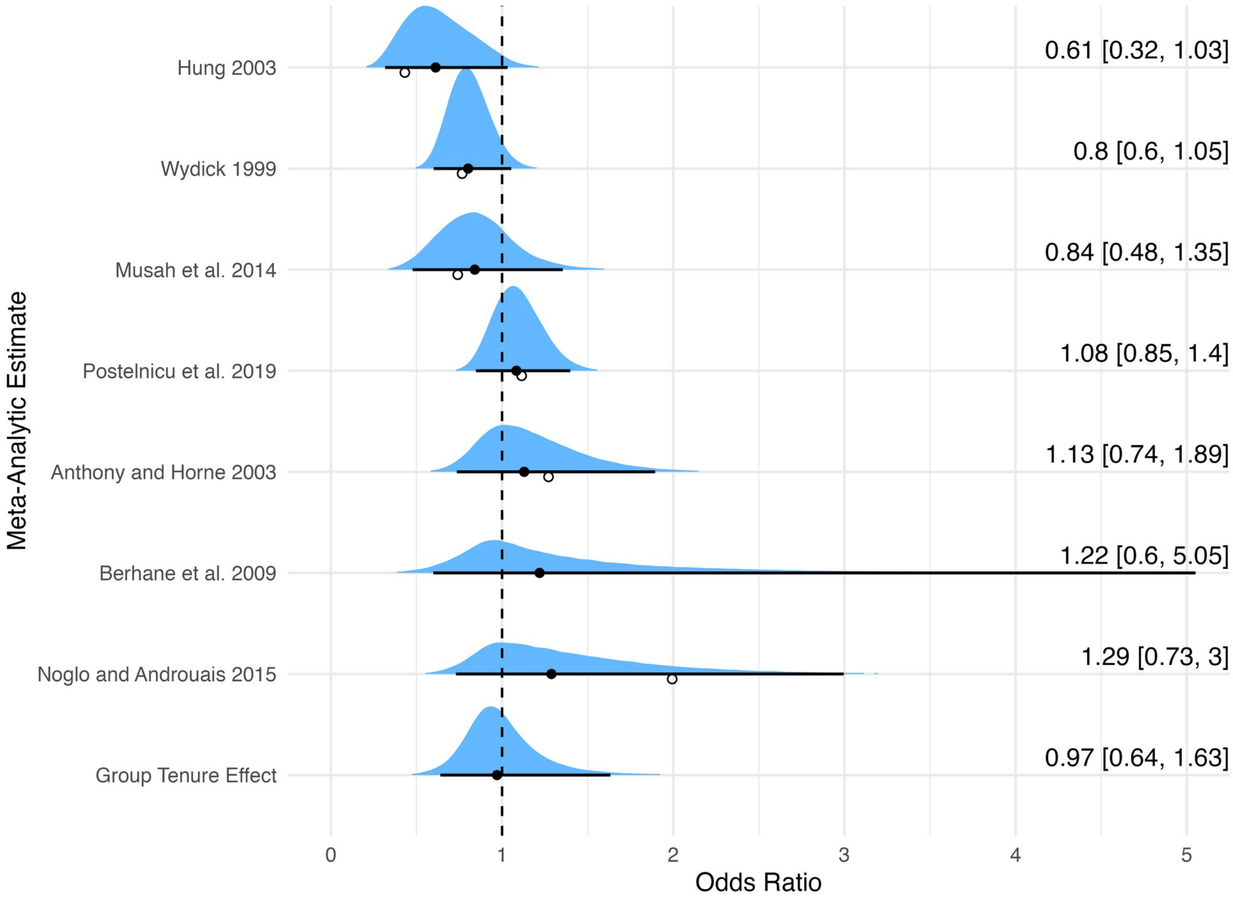

Figure S4. Forest plot for variable VC5 Geographic Proximity

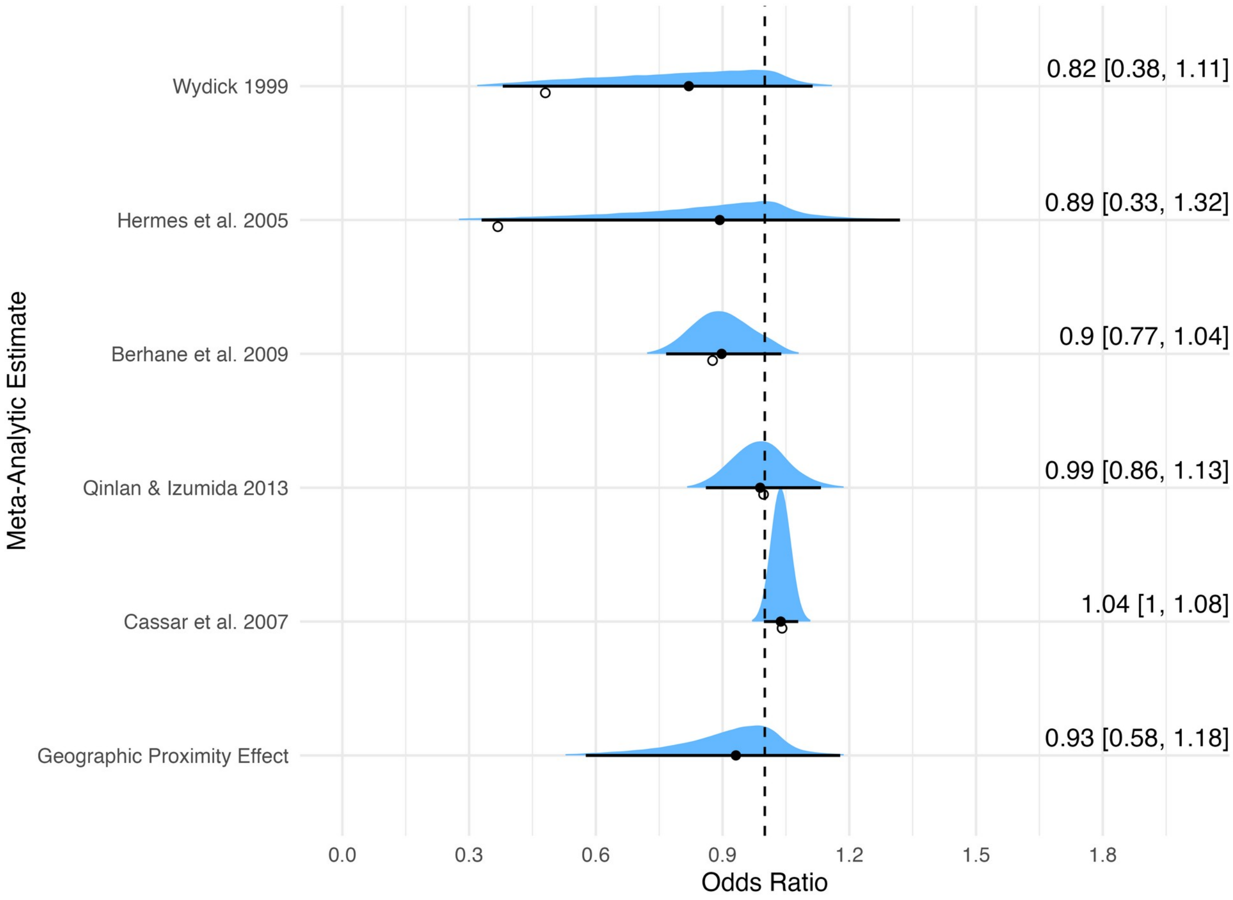

Figure S5. Forest plot for variable VC6 Member Management

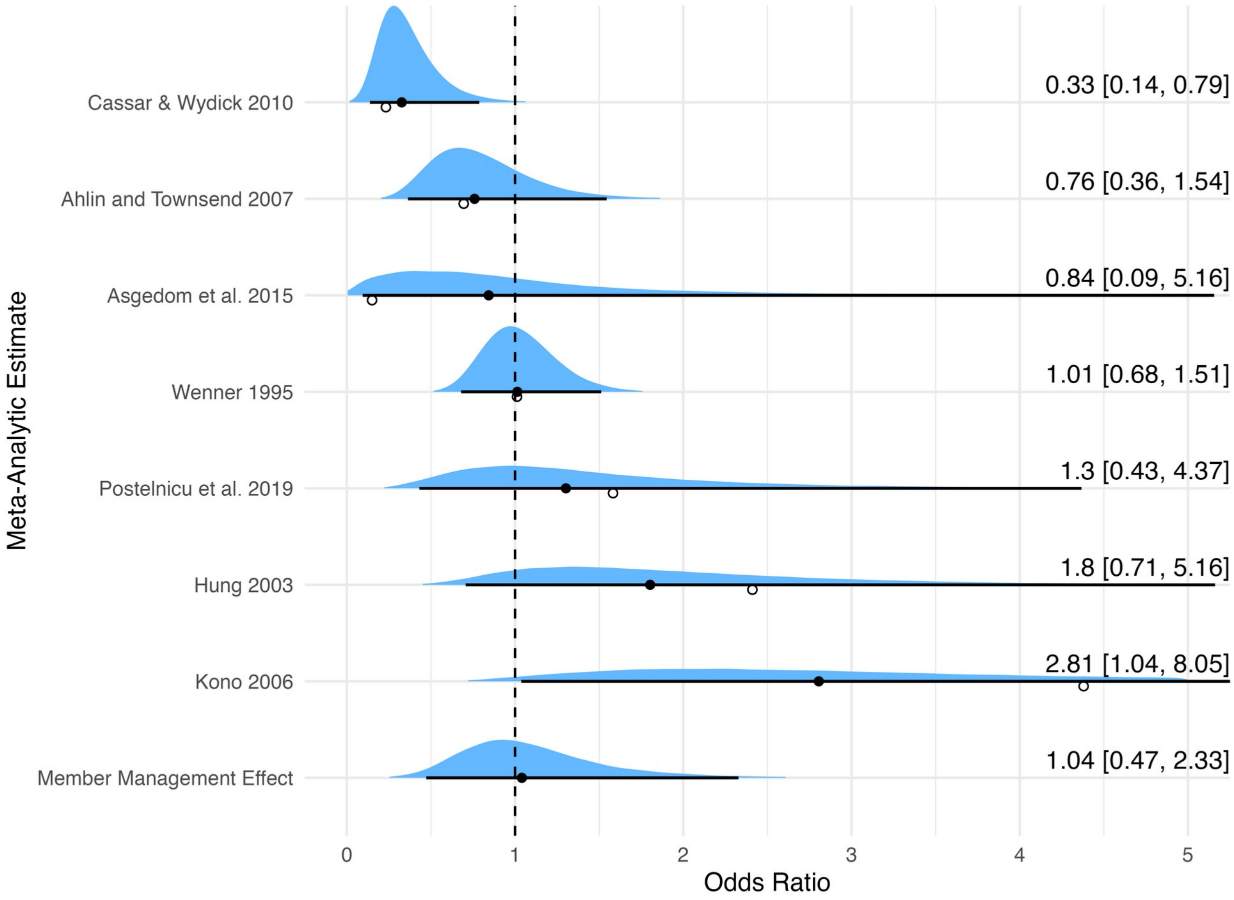

Figure S6. Forest plot for variable VC7 Group Sanctions

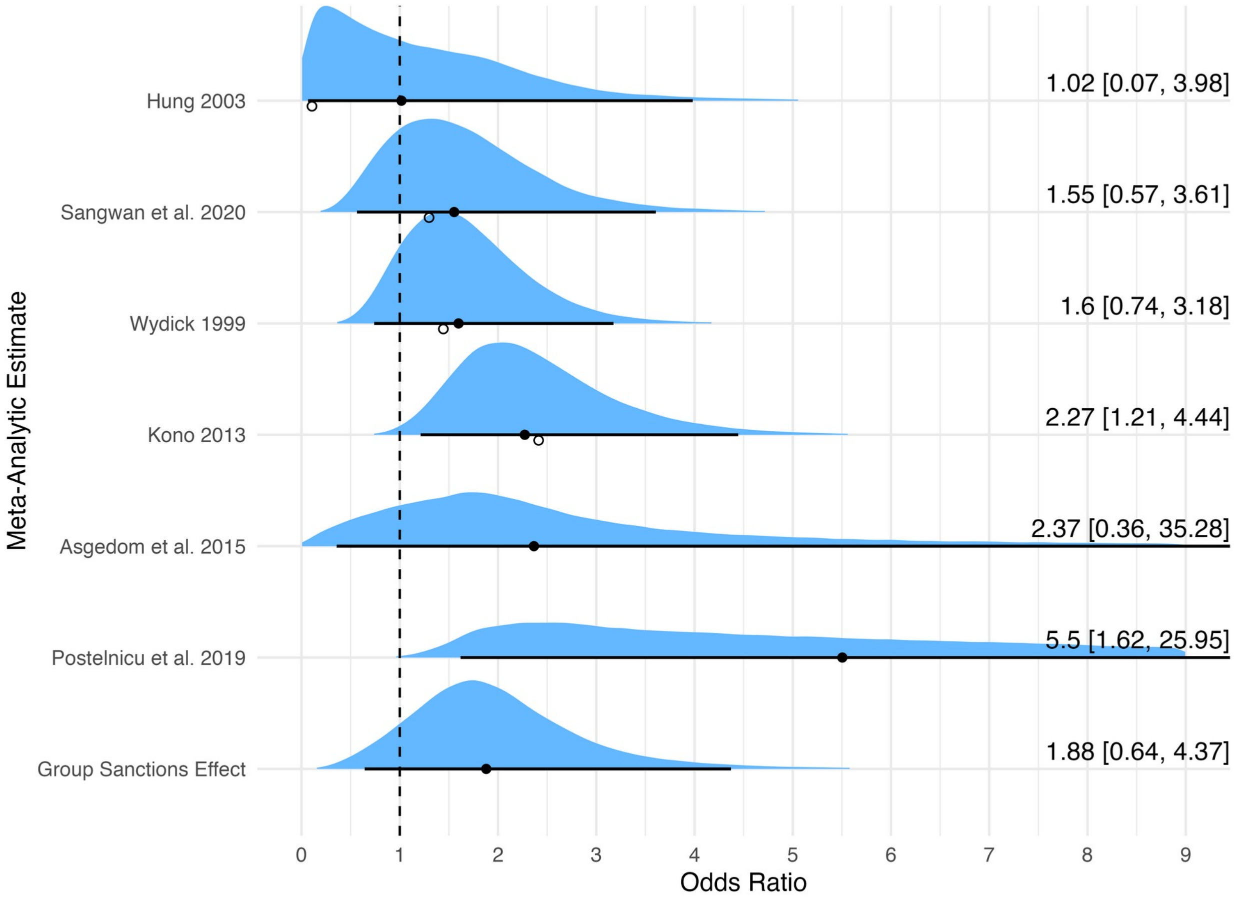

Figure S7. Forest plot for variable VC8 Peer Monitoring

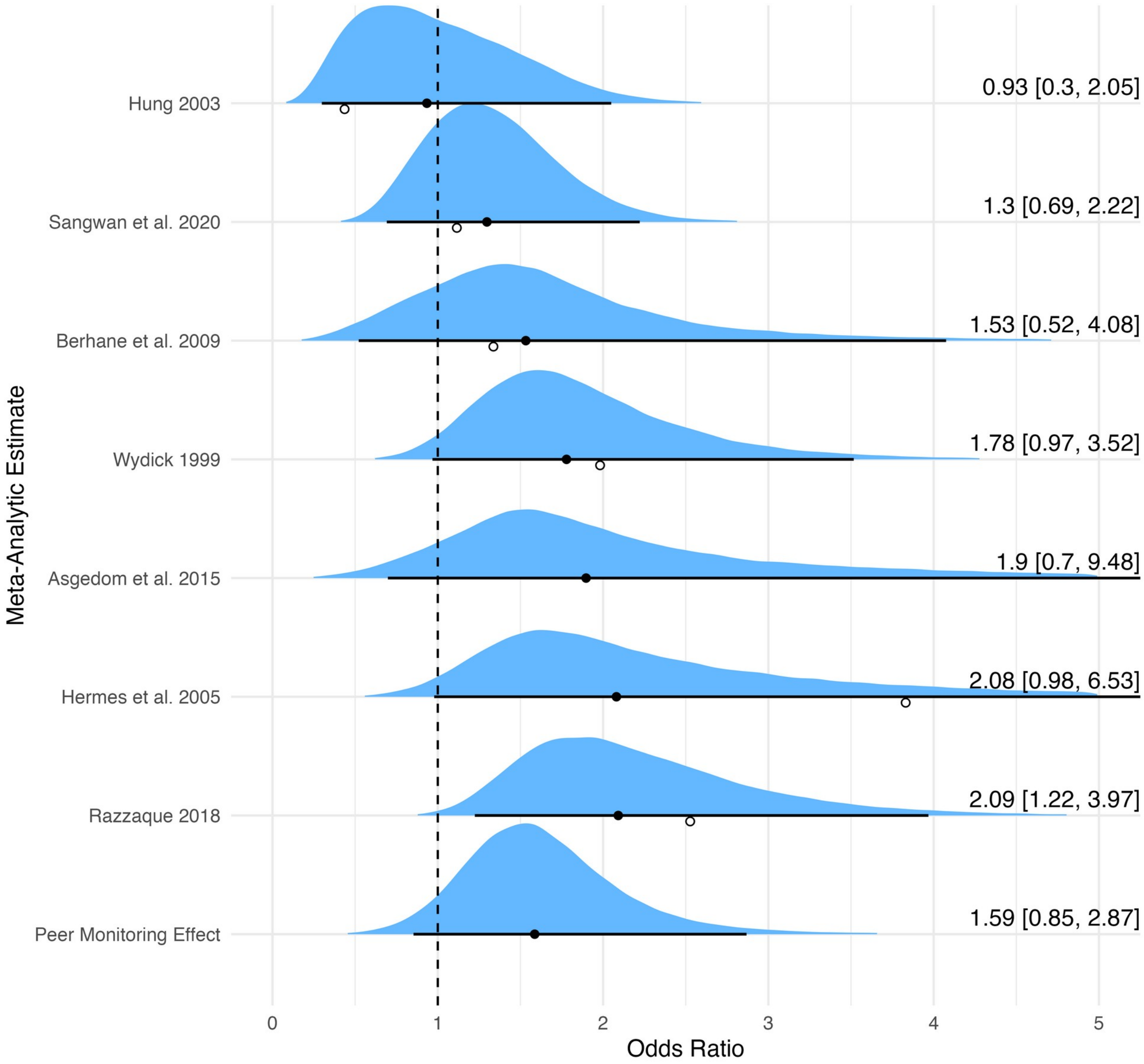

Figure S8. Forest plot for variable VC9 External Monitoring

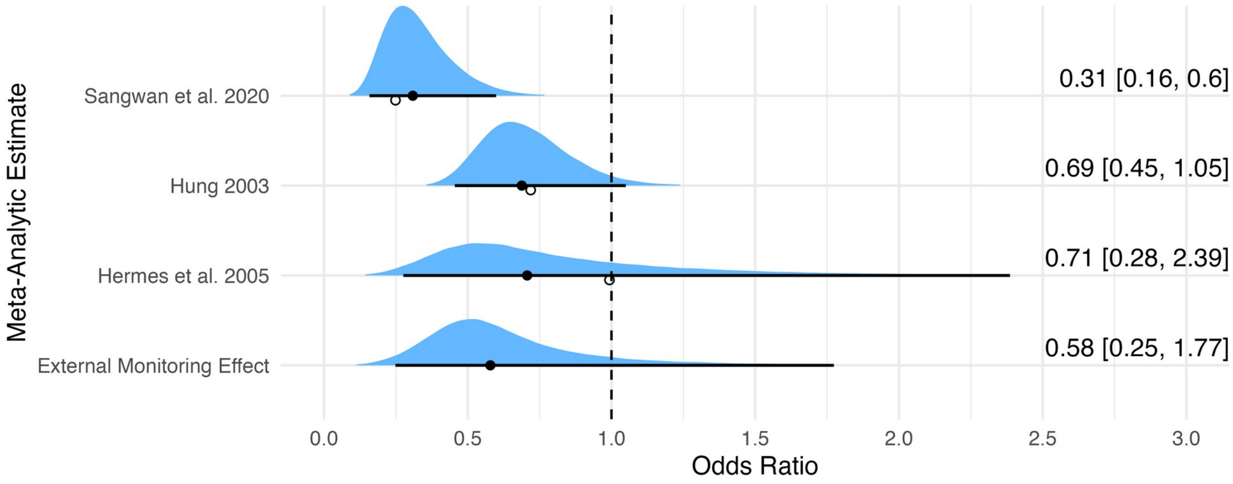

Figure S9. Forest plot for variable VC10 Group Size

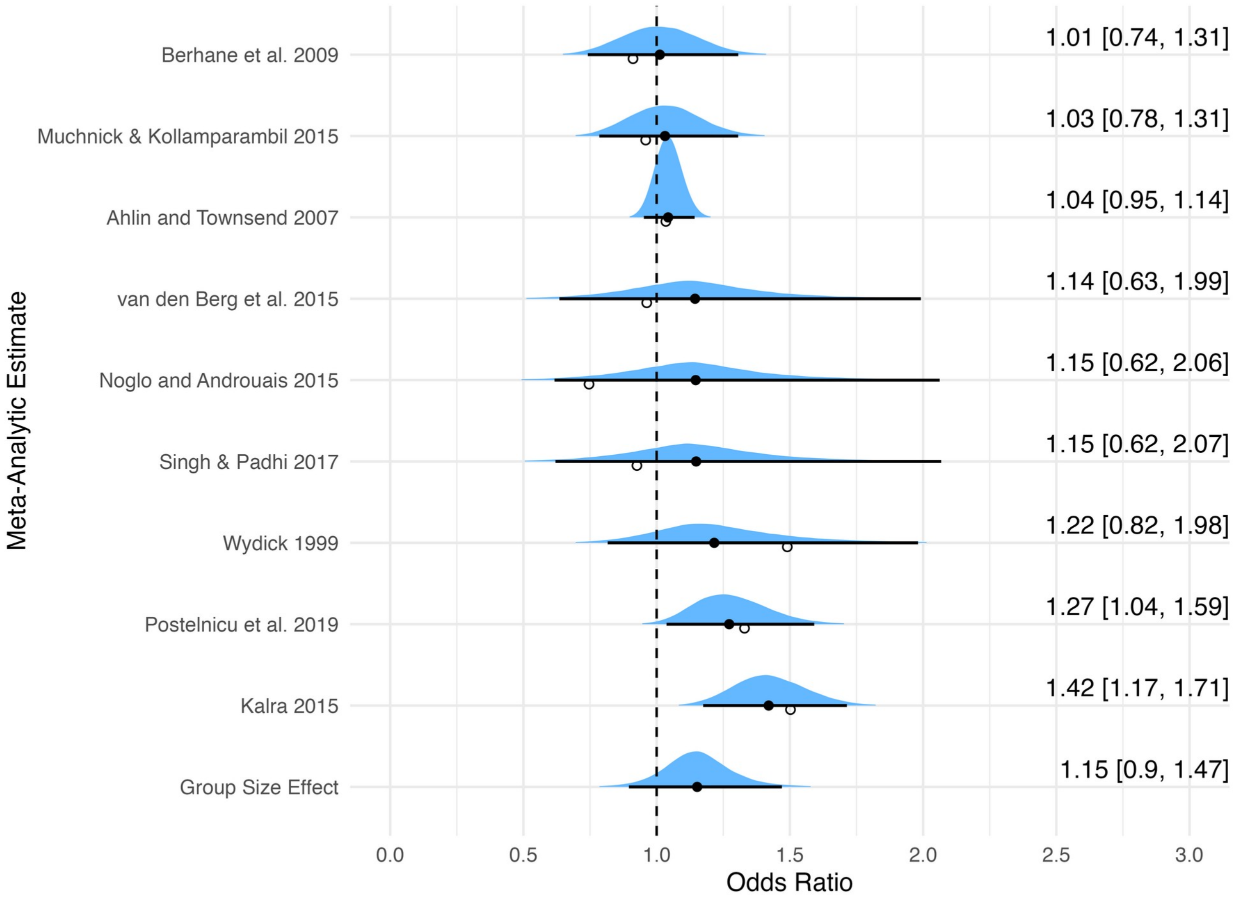

Figure S10. Forest plot for variable VC11 Borrower Age

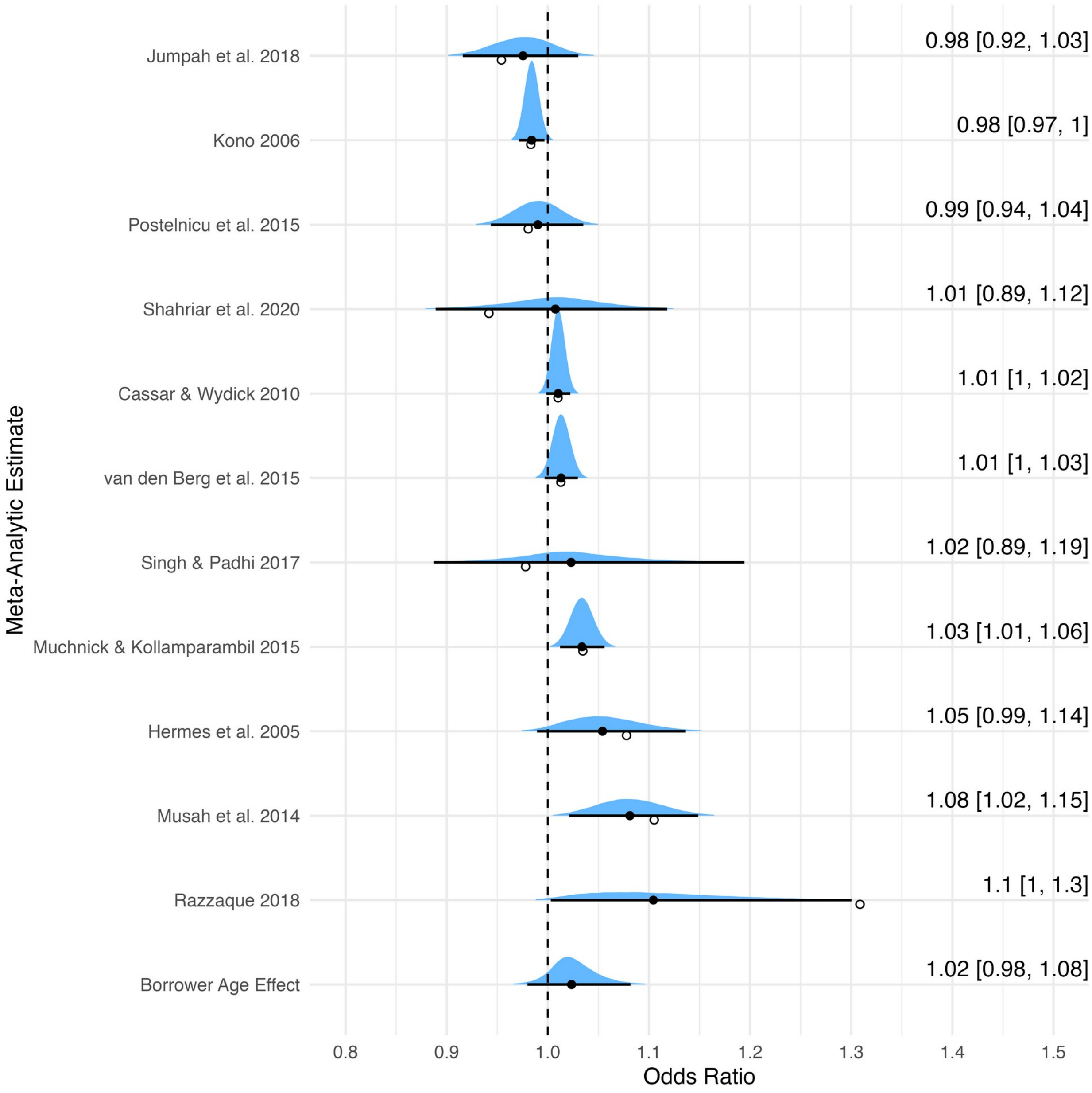

Figure S11. Forest plot for variable VC12 Borrower Sex

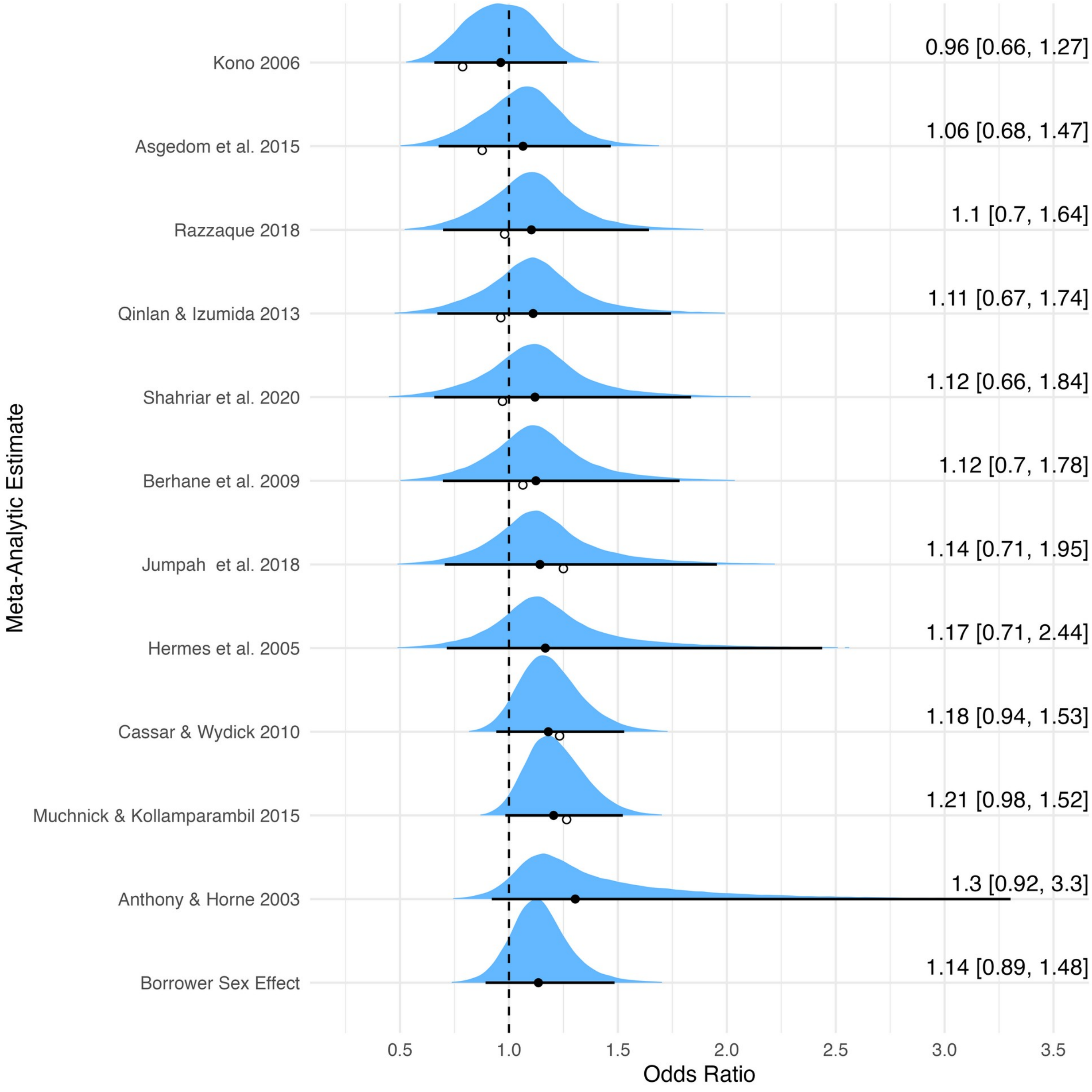

SM8. Publication Bias Analysis

A problem affecting the reliability of meta-analytic effects is that researchers may be more likely to publish studies with results that support their hypotheses, or those that include statistically significant results (Rosenthal, 1979; Sterling, 1959). If studies are repeatedly left unpublished for any reason that is associated with their outcomes, this could result in publication bias, producing a research literature with distorted findings and therefore biased estimation of meta-analytic effects. Because we do not have strong assumptions or good evidence regarding the level of publication bias likely to affect the microfinance loan repayment literature, we will model the potential impact of publication bias with a Bayesian Copas selection model (Copas, 1999; Copas and Shi, 2001), using the *RobustBayesianCopas* package (Bai, 2020) in R.

The Copas selection model assumes the probability of a study being selected (i.e. published) is a function of the size of its effect estimate and the standard error of this estimate. Unlike graph-based or regression test methods, the Copas selection model includes an explicit mechanism linking the publication process with the pattern of observed study estimates (Schwarzer et al., 2010). By calculating the difference between the posterior distribution of our original meta-analytic model, and that of an equivalent Copas selection model, we can estimate the potential impact of publication bias (Bai et al., 2020). To do so we will use Bai's *D* measure (Bai et al., 2020), for which values close to 0 indicate the distributions are nearly identical (suggesting negligible publication bias), and values close to 1 indicate non-overlapping distributions (suggesting severe publication bias).

Table S7. Bai's D measure of publication bias for each variable.

**Variable Category**	**Bai's *D***
Borrower Relatedness	0.07
Prior Acquaintance	0.04
Group Tenure	0.08
Geographic Proximity	0.09
Member Management	0.05
Group Sanctions	0.05
Peer Monitoring	0.08
External Monitoring	0.05
Group Size	0.03
Borrower Age	0.03
Borrower Sex	0.10

Supplementary Materials References

Bai, R. (2020). RobustBayesianCopas: Robust Bayesian Copas Selection Model. https://doi.org/10.32614/CRAN.package.RobustBayesianCopas

Bai, R., Lin, L., Boland, M.R., Chen, Y. (2020). A robust Bayesian Copas selection model for quantifying and correcting publication bias. arXiv preprint arXiv:2005.02930. https://doi.org/10.48550/arXiv.2005.02930

Cheung, M. W.-L. (2014). Modeling dependent effect sizes with three-level meta-analyses: A structural equation modeling approach. *Psychological Methods*, *19*(2), 211–229. https://doi.org/10/gcz6v4

Copas, J. (1999). What works?: selectivity models and meta-analysis. *Journal of the Royal Statistical Society Series* 162, 95–109. https://doi.org/10/fb4m24

Copas, J.B., Shi, J.Q. (2001). A sensitivity analysis for publication bias in systematic reviews. *Statistical Methods in Medical Research 10*, 251–265. https://doi.org/10/gf495n

Davey, J., Turner, R. M., Clarke, M. J., & Higgins, J. P. (2011). Characteristics of meta-analyses and their component studies in the Cochrane Database of Systematic Reviews: A cross-sectional, descriptive analysis. *BMC Medical Research Methodology*, *11*(1), 160. https://doi.org/10/dbsckf

Gehrig, S., Mesoudi, A., & Lamba, S. (2021). Banking on cooperation: An evolutionary analysis of microfinance loan repayment behaviour. *Evolutionary Human Sciences 3, e2.* https://doi.org/10.1017/ehs.2020.64

Higgins, J. P. T., Thompson, S. G., & Spiegelhalter, D. J. (2009). A re-evaluation of random-effects meta-analysis. *Journal of the Royal Statistical Society: Series A (Statistics in Society)*, *172*(1), 137–159. https://doi.org/10/dmthxv

Rosenthal, R., 1979. The file drawer problem and tolerance for null results. *Psychological Bulletin 86*, 638–641. https://doi.org/10/d5sxt3

Schwarzer, G., Carpenter, J., Rücker, G. (2010). Empirical evaluation suggests Copas selection model preferable to trim-and-fill method for selection bias in meta-analysis. *Journal of Clinical Epidemiology 63*, 282–288. https://doi.org/10/fhsz96

Smith, T. C., Spiegelhalter, D. J., & Thomas, A. (1995). Bayesian approaches to random-effects meta-analysis: A comparative study. *Statistics in Medicine*, *14*(24), 2685–2699. https://doi.org/10.1002/sim.4780142408

Spiegelhalter, D. J., Abrams, K. R., & Myles, J. P. (2004). *Bayesian approaches to clinical trials and health care evaluation*. Wiley.

Sterling, T.D. (1959). Publication decisions and their possible effects on inferences drawn from tests of significance—or vice versa. *Journal of the American Statistical Association 54*, 30–34. https://doi.org/10/gckf9z

Sterne, J. A., Hernán, M. A., Reeves, B. C., Savović, J., Berkman, N. D., Viswanathan, M., Henry, D., Altman, D. G., Ansari, M. T., Boutron, I., Carpenter, J. R., Chan, A.-W., Churchill, R., Deeks, J. J., Hróbjartsson, A., Kirkham, J., Jüni, P., Loke, Y. K., Pigott, T. D., … Higgins, J. P. (2016). ROBINS-I: A tool for assessing risk of bias in non-randomised studies of interventions. *BMJ*, *355*. https://doi.org/10/gdf378

Van den Noortgate, W., López-López, J. A., Marín-Martínez, F., & Sánchez-Meca, J. (2013). Three-level meta-analysis of dependent effect sizes. *Behavior Research Methods*, *45*(2), 576–594. https://doi.org/10.3758/s13428-012-0261-6

Vehtari, A., Gelman, A., & Gabry, J. (2017). Practical Bayesian model evaluation using leave-one-out cross-validation and WAIC. *Statistics and Computing*, *27*(5), 1413–1432. https://doi.org/10/gdj2kz

Westreich, D., & Greenland, S. (2013). The Table 2 Fallacy: Presenting and Interpreting Confounder and Modifier Coefficients. *American Journal of Epidemiology*, *177*(4), 292–298. https://doi.org/10.1093/aje/kws412

Williams, D. R., Rast, P., & Bürkner, P.-C. (2018). Bayesian Meta-Analysis with Weakly Informative Prior Distributions. *PsyArXiv*. https://doi.org/10/ggj3rq
